# Sequence Analysis to Phenotype Health Care Patterns in Adults With Musculoskeletal Conditions Using Primary Care Electronic Health Records

**DOI:** 10.1002/acr.25514

**Published:** 2025-04-19

**Authors:** Smitha Mathew, George Peat, Emma Parry, Ross Wilkie, Kelvin P. Jordan, Jonathan C. Hill, Dahai Yu

**Affiliations:** ^1^ Keele University Keele Staffordshire United Kingdom; ^2^ Sheffield Hallam University Sheffield United Kingdom

## Abstract

**Objective:**

The aim of this study was to apply sequence analysis (SA) to phenotype health care patterns of adult patients with musculoskeletal (MSK) conditions using primary care electronic health records and to investigate the association between these health care patterns and patients’ self‐reported outcomes after consultation.

**Methods:**

Data from the Multilevel Integrated Data for musculoskeletal health intelligence and Actions program conducted in North Staffordshire and Stoke‐on‐Trent, United Kingdom, was used. The study included patients aged ≥18 years who consulted primary care for MSK conditions between September 2021 and July 2022. SA was employed to categorize patients with similar health care patterns in primary care in the five years before their index consultation in respect to consultations, analgesic prescriptions, imaging, physiotherapy, and secondary care referrals. Associations of sociodemographic characteristics and self‐reported outcome with clusters were determined.

**Results:**

In total, 1,875 patients consulting primary care for MSK conditions were available for analysis. SA identified five clusters of previous health care patterns among patients with MSK conditions, including “increasing consultation and analgesia” (5.60%), “low consultation and health care use” (57.39%), “high consultation and health care use” (8.32%), “low consultation but high analgesia” (13.01%), and “low consultation but moderate health care use” (15.68%). Patients in the “high consultation and health care use” group were predominantly female, were older, had obesity, had more comorbidities, and lived in the most deprived areas compared to those in the “low consultation and health care use” group. Additionally, self‐reported outcomes varied significantly among clusters, with patients in the “high consultation and health care use” group reporting worse self‐reported outcomes.

**Conclusion:**

This analysis identified five distinct clusters of health care patterns for patients with MSK conditions in primary care and observed substantial variations in patients’ self‐reported outcomes and sociodemographic profiles across these different groups of patients.

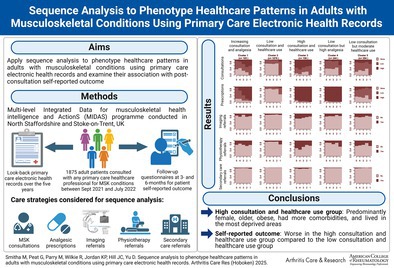

## INTRODUCTION

Musculoskeletal (MSK) conditions are a major cause of pain and disability worldwide. In the United Kingdom, more than 20 million people live with an MSK condition.^1^ MSK conditions are primarily assessed and managed in primary care. It accounts for 12% to 14% of primary care consultations in adults, and a substantial portion of health care expenditure is allocated to managing these conditions.^2^ A range of different interventions are recommended for the management of MSK conditions, including providing advice on self‐management and exercise, referring patients for nonpharmacologic treatments such as physiotherapy, and prescribing analgesics to alleviate pain and symptoms.^3^

**SIGNIFICANCE & INNOVATIONS**

- Our study identified five distinct patterns of health care utilization in primary care among adult patients with musculoskeletal conditions using sequence analysis.
- We observed inequalities in health care utilization patterns based on patients’ characteristics and significant variations in patients’ self‐reported outcomes across different clusters of health care utilization patterns. Specifically, patients from socioeconomically deprived areas who were predominantly older and female and who had obesity and multiple comorbidities showed higher consultation rates, higher health care use, and poorer short‐term outcome.
- These findings highlighted the importance of addressing disparities in health care access and the need for targeted interventions for patients at risk of poorer health outcomes.

Pain associated with MSK conditions leads to high health care use, and patients seeking health care may find themselves consulting a diverse range of health care professionals and receiving a mix of analgesic prescriptions, imaging, physiotherapy, and secondary care referrals.^4^, ^5^ Understanding patterns within these interactions can provide insights into how different patient subgroups use health care services. By analyzing these patterns, health care providers can identify the specific needs of patient subgroups. It enables health care planners to allocate resources more strategically, ensuring that they are directed to where they are most needed. Moreover, a comprehensive understanding of care patterns helps identify service gaps and areas for improvement. This knowledge allows for the optimization of health care delivery by addressing disparities in service utilization. Overall, it supports identifying specific health care needs, informs strategic resource allocation, and contributes to improving health care delivery and patient outcomes.^6^, ^7^

Patients’ self‐reported outcome measures are valuable for evaluating perceptions of health, symptoms, and the effectiveness of MSK management.^8^ These measures capture information primarily focusing on pain levels, activity limitations, and overall quality of life rather than clinical measures.^9^ Several studies have highlighted an association between chronic pain and increased health care utilization.^10^, ^11^, ^12^ Additionally, a correlation has been observed between low health‐related quality of life and high health care utilization.^12^ Evidence from a primary care prospective observational cohort study further indicates that subgroups of individuals with different levels of risk for poor MSK pain outcomes exhibit different levels of health care utilization.^13^ Relating health care utilization patterns to patients’ self‐reported outcomes might direct attention toward potentially poorly targeted or ineffective patterns of care.

In recent years, sequence analysis (SA) has emerged as a promising analytical approach in health care research due to its ability to uncover valuable insights and patterns from real‐world data.^14^ SA is used to analyze ordered sets of data, often referred to as sequences. This method is commonly used in social science to identify patterns in life course trajectories and to study transitions into adulthood^15^, ^16^ or career patterns^17^ by examining longitudinal data representing events experienced by individuals over time. In health care, SA allows researchers to analyze sequences of medical events, such as diagnoses, treatments, and procedures, to understand disease progression and care pathways.^6^, ^18^, ^19^ SA enables the exploration of health care utilization patterns, including patient journeys through the health care system, patterns of service utilization, and transitions between different levels of care.^20^, ^21^, ^22^

A conventional SA involves three steps: defining events as sequences of successive categorical states, calculating dissimilarities between pairs of sequences, and building a typology of the sequences.^23^ The states in the sequence should be clinically meaningful and relevant to the research objectives. Dissimilarity is a quantitative measure indicating the degree to which two individuals followed distinct sequences. There are different dissimilarity measures based on alignment and nonalignment techniques. The choice of dissimilarity measure may affect the results of SA; therefore, researchers select an appropriate measure aligned with their research objective.^24^ Finally, a cluster analysis is performed to classify individuals with similar sequences.

In this study, we focus on the identification of different health care patterns among adult patients with MSK conditions in primary care more than five years before their index consultation, as well as examining the effect of these patterns on patients’ self‐reported outcomes. By examining historic care patterns, we can comprehensively understand the various treatment strategies patients have experienced, which might influence their current health status and outcome.

Therefore, the primary objective of this study was to apply SA to phenotype health care patterns of patients with MSK conditions from routinely collected primary care electronic health records (EHRs). The secondary objective was to investigate the association between the identified health care patterns and patients’ self‐reported outcomes after consultation.

## MATERIALS AND METHODS

### Data source and population

The Multilevel Integrated Data for musculoskeletal health intelligence and Actions (MIDAS) program, funded by the Nuffield Foundation and Versus Arthritis, aims to develop a comprehensive, place‐based system for MSK health data in North Staffordshire and Stoke‐on‐Trent, United Kingdom. MIDAS‐GP is one observational cohort study within the overall MIDAS project, and is designed to collect, link, and explore data from patient‐report, electronic health records, and other sources for adults presenting with common, painful MSK conditions presenting in general practiced (GP). The study focuses on integrating data from various clinical settings to enhance MSK care pathways. The prespecified MIDAS‐GP study protocol is available at Open Science Framework (https://osf.io/e542w/). The study received ethical approval from Yorkshire & The Humber‐Leeds West Research Ethics Committee (Reference: 21/YH/0178).

The eligible participants for this study included patients aged 18 years and older, registered with 30 participating general practices and who consulted any primary care health care professional within the practice for a painful, noninflammatory MSK condition. Recruitment was conducted from September 2021 to July 2022, staggered across different practices, with recruitment periods lasting from three to six months. Relevant MSK pain‐related consultations were identified using a pre‐specified Systematized Nomenclature of Medicine Clinical Terms (SNOMED CT) code list (Supplementary Table S1). Eligible participants were invited to complete a baseline questionnaire on MSK health and care and were asked for their consent to link the questionnaire to EHRs. The consenting participants were further asked to complete the follow‐up questionnaires at three and six months.

The information on patient's demographic, socioeconomic, comorbidities, and MSK management strategies were derived from the primary care EHR in the five years before index MSK consultation. The list of comorbidities used was produced after cross‐mapping morbidities in National Institute for Health and Care Excellence (NICE) multimorbidity indicator for general practice,^25^ Charlson,^26^ and Elixhauser^27^ comorbidity indices, and potentially relevant case‐mix adjustment methods.^28^ Comorbidity code lists are available at Open Science Framework (https://osf.io/e542w/). The MSK management information included MSK‐related primary care consultations, relevant prescriptions for medications, referrals for imaging (eg, radiographs, magnetic resonance imaging, or CT scans), referrals for physiotherapy, and referrals for secondary care (MSK triage, rheumatology, trauma, and orthopedic departments). Patients’ neighborhood deprivation was also considered. We used the English index of multiple deprivation (IMD) 2019 rank as a composite measure of neighborhood deprivation, which covers seven domains of material deprivation including income, employment, education and skills training, health deprivation and disability, barriers to housing and services, crime, and living environment.^29^ The IMD classifies the areas into five quintiles based on relative disadvantage, with quintile 1 being the most deprived and quintile 5 being the least deprived. Additionally, patients’ MSK Health Questionnaire (HQ) scores at baseline, three months, and six months after index consultation were considered. The MSK‐HQ is a 14‐item questionnaire that captures key outcomes that patients with MSK conditions have prioritized as important for use across clinical pathways.^30^ Scores range from 0 to 56, with higher scores indicating better MSK health over the past two weeks.^30^ The data of this study are available upon request.

### Statistical analysis

To explore the patterns of utilization of key MSK management strategies in primary care, we employed a multichannel SA involving five domains: MSK‐related consultations, analgesic prescriptions, imaging referrals, physiotherapy referrals, and secondary care referrals. The primary step in SA was defining the states within the sequence, the observation period, and the time unit. The health care patterns of patients with MSK conditions were observed for five years before their index consultation. The MSK management information was retrieved as annual count data. So, we defined three categorical states for consultations and analgesic prescriptions: “none,” “low,” and “high,” representing zero, one to three, and four or more instances, respectively, and two categorical states for imaging, physiotherapy, and secondary care referrals: “no” and “yes” occurrence during the year (detailed in the Supplementary Material S1). If the care event is not recorded in the system, it is considered to have not occurred. We defined care sequences for each domain for each patient, with each sequence consisting of five states (one for each year).

For the analysis of sequences, we chose optimal matching (OM) edit distance, the most often used approach to measure the dissimilarity between pairs of sequences.^14^ OM measures the dissimilarity between two sequences by determining the minimum cost required to transform one sequence into another by edit operations such as insertion, deletion, or substitution of states. We opted for a data‐driven cost for insertion/deletion, and substitutions based on the frequency of the states in the sequences, referred to as INDELSLOG. In this approach, insertion/deletion costs were calculated initially as the logarithm of the inverse of the relative frequency of the states, as log(2/[1 + f]), in which “f” is the relative frequency of the states. Then, the substitution costs between the two states are computed by summing their insertion/deletion costs.^31^ The rationale behind this approach is that inserting or deleting rare states is more costly than inserting or deleting frequent states, and substituting rarely observed states costs more than substituting common states.^31^ The multidomain dissimilarity matrix was computed by adding the domain‐specific dissimilarity matrixes. Based on the computed dissimilarity matrix, we performed an agglomerative hierarchical cluster analysis with Ward's linkage to classify patients with similar care patterns. The optimum number of clusters was determined based on the dendrogram, inertia jump curve, cluster quality indices, and clinical relevance and interpretability (explanation of the selection criteria is given in the Supplementary Material S2). To visualize the care patterns, we used sequence index plots and state distribution plots provided by the SA. State distribution plot shows the distribution of states for each time unit, whereas each line in the sequence index plot represents an individual sequence.^24^

We compared patients’ demographic and health characteristics among the derived clusters using the chi‐square test, *t*‐test, and analysis of variance. A multinomial logistic regression model was used to assess the association between patients’ profiles and cluster membership. A linear mixed model was used to test the difference in patient‐reported MSK‐HQ scores between clusters. The model included the fixed, categorical effects of cluster, time, cluster‐by‐time interaction, sex, comorbidities, and IMD, alongside continuous, fixed covariates for age and body mass index (BMI). To account for within‐participant variability, an unstructured covariance structure was applied to model the within‐participant errors. The missing data in BMI (n = 280) were imputed by multiple imputation using chained equations.^32^ Sensitivity analyses were conducted to ensure the reproducibility of the results. For this, patients with MSK conditions were subgrouped into those with osteoarthritis (OA) and those with low back pain (LBP), and the SA was repeated within these subgroups. The SA was conducted using the TraMineR and WeightedCluster packages in R, and all other analyses were performed using Stata version 18 (StataCorp).

## RESULTS

### Participants

A total of 2,008 patients (14.9%) responded at baseline, of whom 1,875 patients consented and were successfully linked to their EHRs and hence form the primary population for analysis (detailed in Supplementary Figure S1). Among these patients, the mean ± SD age was 57.74 ± 15.50 years, and the mean ± SD BMI was 29.18 ± 6.91. Female participants accounted for 65.76% of the patients, 32.43% were classified as obese, and 28.27% were from the most deprived areas (Table 1). Patients’ care sequences of each domain were presented in sequence index plots (Supplementary Figure S2).

**Table 1:** Patients’ baseline characteristics*

Variables	Patients (N = 1,875)
Sex, n (%)	
Female	1,233 (65.76)
Male	642 (34.24)
Age, mean (SD)	57.74 (15.50)
Age group, n (%)	
18–34 y	157 (8.37)
35–44 y	234 (12.48)
45–54 y	362 (19.31)
55–64 y	430 (22.93)
65–74 y	407 (21.71)
75–84 y	241 (12.85)
85+ y	44 (2.35)
BMI, mean (SD)	29.18 (6.91)
BMI, n (%)	
Underweight (<18.5)	26 (1.39)
Normal (18.5–24.9)	399 (21.28)
Overweight (25–29.9)	562 (29.97)
Obese (≥30)	608 (32.43)
Missing	280 (14.93)
Comorbidity count, n (%)	
0	829 (44.21)
1	577 (30.77)
2	329 (17.55)
3+	140 (7.47)
Index of multiple deprivation, n (%)	
Quintile 1 (most deprived)	530 (28.27)
Quintile 2	383 (20.43)
Quintile 3	398 (21.23)
Quintile 4	320 (17.07)
Quintile 5 (least deprived)	244 (13.01)
Race and ethnicity, n (%)	
White	1,788 (95.36)
Asian	31 (1.65)
Mixed	11 (0.59)
Black	28 (1.49)
Other	17 (0.91)

* BMI, body mass index.

### SA

**Figure 1:**
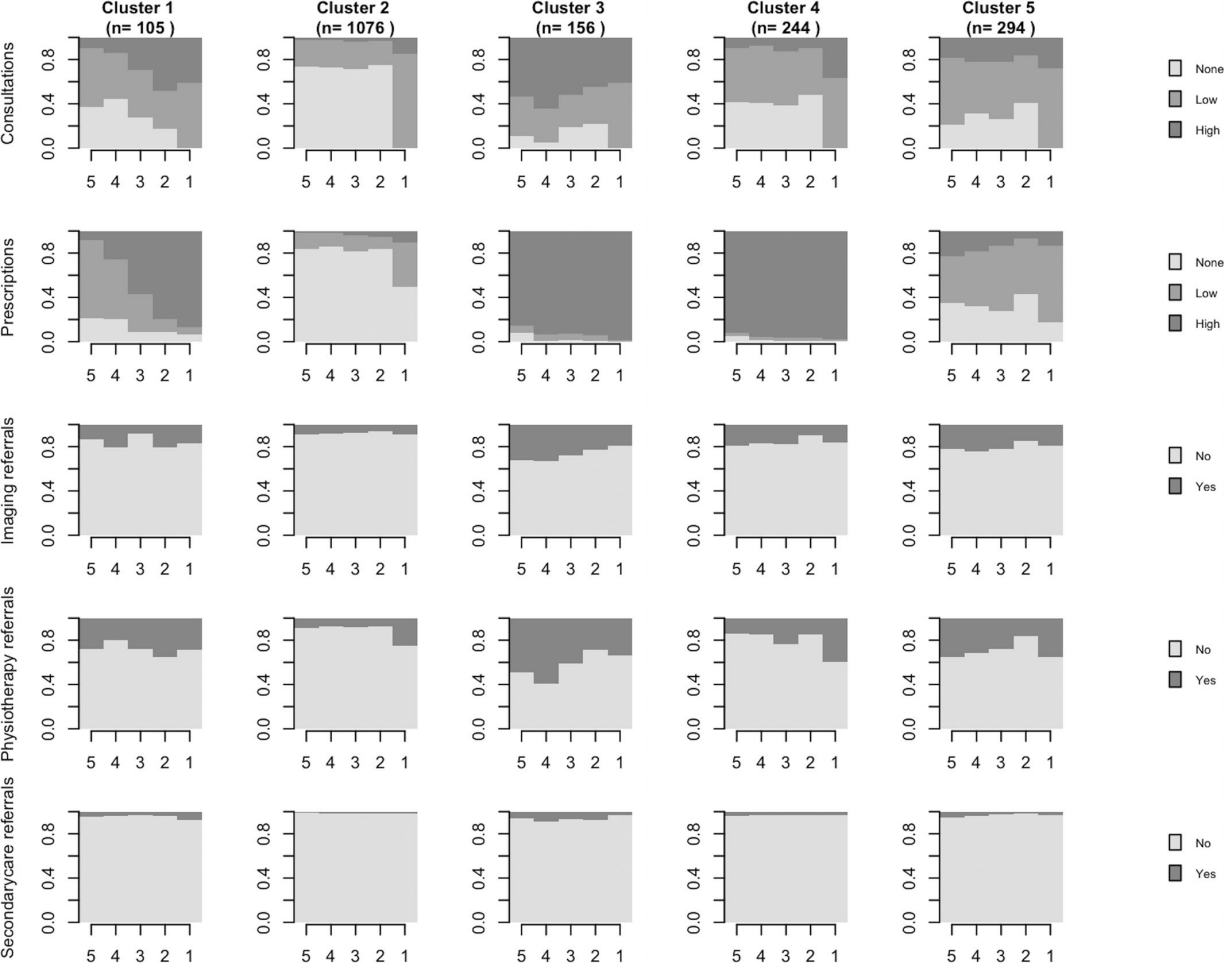
State distribution plot of care sequence typology by domain (consultations, prescriptions, imaging, physiotherapy, and secondary care referrals).

By the multichannel SA of the domains—MSK consultations, analgesic prescriptions, imaging referrals, physiotherapy referrals, and secondary care referrals—patients with similar care sequences were classified into five distinct clusters (Figure 1) based on the dendrogram, inertia jump curve, and cluster quality indices (Supplementary Table S2 and Supplementary Figures S3 and S4). The characteristics of the identified clusters are as follows:

- Cluster 1 (n = 105, 5.60%) patients were characterized by a marked increase in high‐level (ie, four or more) consultations and analgesic prescriptions over the five years, accompanied by moderate imaging and physiotherapy, and minimum secondary care referrals. This cluster can be labeled as “increasing consultation and analgesia.”
- Cluster 2 (n = 1,076, 57.39%) consisted of patients with low‐level (one to three) consultations and analgesic prescriptions mainly in the index year, and minimal imaging, physiotherapy, and secondary care referrals. This cluster can be labeled as “low consultation and health care use.”
- Cluster 3 (n = 156, 8.32%) was made up of patients with consistently higher levels of consultation, analgesic prescriptions, imaging, physiotherapy, and secondary care referrals. This cluster can be labeled as “high consultation and health care use.”
- Cluster 4 (n = 244, 13.01%) included patients with low‐level (one to three) consultations, low imaging, physiotherapy, and secondary care referrals, but having higher levels (four or more) of analgesic prescriptions over the five years. This cluster can be labeled as “low consultation but high analgesia.”
- Cluster 5 (n = 294, 15.68%) consisted of patients with low‐levels (one to three) of consultations, analgesic prescriptions, and secondary care referrals, but moderate levels of imaging and physiotherapy referrals. This cluster can be labeled as “low consultation but moderate health care use.”

### Potential predictors of cluster membership

Patients’ characteristics by clusters of similar care patterns were presented in Supplementary Table S3. Table 2 shows the findings of the multinomial logistic regression model computed to examine potential predictors of the identified clusters. Odds ratios were calculated to indicate the likelihood of being in a particular cluster compared to the reference cluster. The reference cluster used in the analysis was “low consultation and health care use.” Female patients were significantly more likely to be in “high consultation and health care use,” “low consultation but high analgesia,” and “low consultation but moderate health care use” clusters, as compared to being in the “low consultation and health care use” cluster. Additionally, older age, obesity, a higher comorbidity index, and socioeconomic deprivation (most deprived) were identified as significant predictors for membership in the “increasing consultation and analgesia,” “high consultation and health care use,” “low consultation but high analgesia,” and “low consultation but moderate health care use” clusters.

**Table 2:** Multinomial logistic regression model for association between patients’ characteristics and different clusters*

Patient characteristic	Clusters of similar care sequences			
	Increasing consultation and analgesia, OR (95% CI)	High consultation and health care use, OR (95% CI)	Low consultation but high analgesia, OR (95% CI)	Low consultation but moderate health care use, OR (95% CI)^b^
Sex				
Male	1	1	1	1
Female	1.51 (0.97–2.35)	2.55 (1.69–3.88)^a^	1.79 (1.30–2.46)^a^	1.87 (1.40–2.51)^a^
Age group				
18–34 y	1	1	1	1
35–44 y	0.62 (0.23–1.69)	1.24 (0.43–3.57)	2.98 (0.96–9.21)	1.41 (0.75–2.62)
45–54 y	1.71 (0.76–3.84)	2.82 (1.10–7.21)^a^	5.73 (1.97–16.66)^a^	1.80 (0.99–3.24)
55–64 y	1.16 (0.50–2.69)	3.50 (1.39–8.82)^a^	7.04 (2.45–20.21)^a^	1.87 (1.04–3.33)^a^
65–74 y	1.96 (0.85–4.52)	5.31 (2.09–13.49)^a^	11.66 (4.06–33.53)^a^	2.79 (1.55–5.01)^a^
75–84 y	2.73 (1.10–6.77)^a^	9.05 (3.41–24.03)^a^	18.99 (6.46–55.84)^a^	3.20 (1.68–6.11)^a^
85+ y	1.69 (0.32–8.89)	8.42 (2.23–31.80)^a^	12.51 (3.26–47.94)^a^	4.31 (1.65–11.26)^a^
BMI				
Underweight/normal (<25)	1	1	1	1
Overweight (25–29.9)	0.98 (0.52–1.84)	1.04 (0.59–1.84)	1.18 (0.77–1.80)	1.22 (0.84–1.76)
Obese (≥30)	2.03 (1.15–3.59)^a^	2.54 (1.52–4.25)^a^	1.79 (1.18–2.71)^a^	1.80 (1.25–2.59)^a^
Comorbidity count				
0	1	1	1	1
1	1.74 (1.07–2.83)^a^	1.58 (0.97–2.56)	2.19 (1.51–3.18)^a^	1.34 (0.99–1.82)
2	1.93 (1.07–3.47)^a^	4.38 (2.72–7.05)^a^	3.90 (2.60–5.85)^a^	1.28 (0.86–1.91)
3+	3.49 (1.65–7.38)^a^	6.65 (3.60–12.28)^a^	5.55 (3.21–9.61)^a^	2.07 (1.19–3.61)^a^
Index of multiple deprivation				
Quintile 1 (most deprived)	1.42 (0.71–2.82)	2.65 (1.34–5.23)^a^	2.09 (1.21–3.62)^a^	1.22 (0.78–1.90)
Quintile 2	1.24 (0.61–2.52)	2.33 (1.16–4.65)^a^	1.84 (1.05–3.22)^a^	1.11 (0.70–1.76)
Quintile 3	0.70 (0.33–1.49)	0.78 (0.36–1.66)	1.30 (0.75–2.26)	0.85 (0.55–1.35)
Quintile 4	0.86 (0.40–1.83)	1.46 (0.70–3.02)	1.34 (0.76–2.38)	0.86 (0.53–1.39)
Quintile 5 (least deprived)	1	1	1	1

* BMI, body mass index; CI, confidence interval; OR, odds ratio.

^a^ These are significant results.

^b^ The reference cluster is low consultation and healthcare use.

### The effect of health care patterns and patients’ MSK‐HQ scores

**Figure 2:**
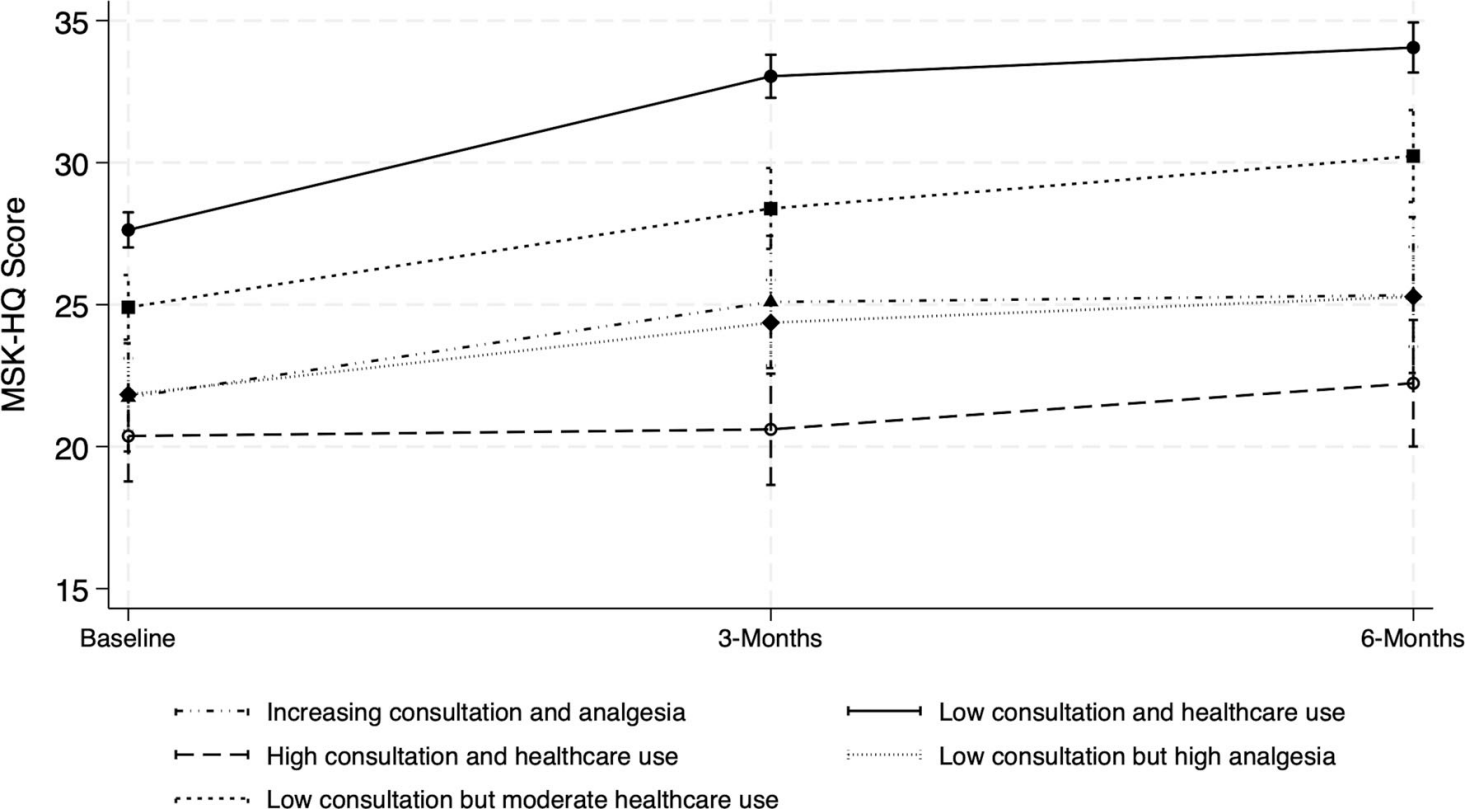
Predicted values of MSK‐HQ score among the distinct clusters. Predicted values were controlled for sex, age, body mass index, comorbidity count, and index of multiple deprivation. MSK‐HQ, Musculoskeletal Health Questionnaire.

Table 3 presents the adjusted estimates for the association between health care patterns and MSK‐HQ score. Figure 2 illustrates the predicted mean MSK‐HQ score values among different clusters at index consultation (baseline), and at three months and six months following the index consultation. The mean patient‐reported MSK‐HQ score was significantly lower (worse MSK health) in the “increasing consultation and analgesia,” “high consultation and health care use,” “low consultation but high analgesia,” and “low consultation but moderate health care use” clusters compared to the “low consultation and health care use” cluster at baseline, three months, and six months; the estimated differences in mean score are presented in Table 4. Additionally, the MSK‐HQ score over time, as indicated by the interaction terms of clusters with similar care sequences and time, showed significantly less improvement at month 3 in the “high consultation and health care use” (coefficient −5.18 [95% confidence interval (CI) −6.92 to −3.43]), “low consultation but high analgesia” (coefficient −2.88 [95% CI −4.28 to −1.49]), and “low consultation but moderate health care use” (coefficient −1.93 [95% CI −3.30 to −0.57]) clusters compared to the improvement in the “low consultation and health care use” cluster. Similarly, less improvement was observed at month 6 in the “increasing consultation and analgesia” (coefficient −2.82 [95% CI −5.27 to −0.37]), “high consultation and health care use” (coefficient −4.57 [95% CI −6.55 to −2.58]), and “low consultation but high analgesia” (coefficient −2.98 [95% CI −4.61 to −1.35]) clusters (Table 3).

**Table 3:** Longitudinal linear mixed model to assess association between clusters of similar care sequence and MSK‐HQ score*

	MSK‐HQ Score
Fixed effects, coefficient (95% CI)	
Intercept	26.19 (23.04 to 29.33)^a^
Cluster of similar care sequences, coefficient (95% CI)	
Increasing consultation and analgesia	−5.90 (−7.90 to −3.89)^a^
High consultation and healthcare use	−7.26 (−9.01 to −5.51)^a^
Low consultation but high analgesia	−5.79 (−7.24 to −4.35)^a^
Low consultation but moderate healthcare use	−2.73 (−4.03 to −1.43)^a^
Time, coefficient (95% CI)	
3 mo	5.41 (4.78 to 6.04)^a^
6 mo	6.42 (5.69 to 7.16)^a^
Interaction terms cluster of similar care sequence x time, coefficient (95% CI)	
Increasing consultation and analgesia x 3 mo	−2.05 (−4.12 to 0.02)
Increasing consultation and analgesia x 6 mo	−2.82 (−3.30 to −0.57)^a^
High consultation and healthcare use x 3 mo	−5.18 (−6.92 to −3.43)^a^
High consultation and healthcare use x 6 mo	−4.57 (−6.55 to −2.58)^a^
Low consultation but high analgesia x 3 mo	−2.88 (−4.28 to −1.49)^a^
Low consultation but high analgesia x 6 mo	−2.98 (−4.61 to −1.35)^a^
Low consultation but moderate healthcare use x 3 mo	−1.93 (−3.30 to −0.57)^a^
Low consultation but moderate healthcare us x 6 mo	−1.10 (−2.65 to 0.45)
Random effects, SD	
Intercept	7.39
Time	2.76

* The reference cluster is low consultation and health care use. Model was controlled for sex, age, body mass index, comorbidity count, and index of multiple deprivation. CI, confidence interval; MSK‐HQ, Musculoskeletal Health Questionnaire.

^a^ These are significant results.

**Table 4:** Difference in MSK‐HQ score from low consultation and health care use at baseline, three months, and six months*

	MSK‐HQ score		
	Baseline, difference (95% CI)	3 mo, difference (95% CI)	6 mo, difference (95% CI)
Increasing consultation and analgesia	−5.90 (−7.91 to −3.89)^a^	−7.95 (−10.40 to −5.49)^a^	−8.72 (−11.61 to −5.83)^a^
Low consultation and health care use	0	0	0
High consultation and health care use	−7.26 (−9.01 to −5.51)^a^	−12.43 (−14.56 to −10.31)^a^	−11.82 (−14.24 to −9.40)^a^
Low consultation but high analgesia	−5.79 (−7.24 to −4.35)^a^	−8.68 (−10.39 to −6.97)^a^	−8.78 (−10.76 to 6.79)^a^
Low consultation but moderate health care use	−2.73 (−4.03 to −1.43)^a^	−4.66 (−6.28 to −3.04)^a^	−3.82 (−5.67 to −1.98)^a^

* Model was controlled for sex, age, body mass index, comorbidity count, and index of multiple deprivation. CI, confidence interval; MSK‐HQ, Musculoskeletal Health Questionnaire.

^a^ These are significant results.

### Sensitivity analysis

To test the reproducibility of the results, two additional SAs were performed by subgrouping the patients with conditions into those with OA and those with LBP. Agglomerative hierarchical cluster analysis with OM and INDELSLOG cost produced five clusters for patients with OA (Supplementary Figure S5), which were similar to the results obtained for patients with MSK conditions. Similarly, the analysis of patients with LBP also resulted in five clusters (Supplementary Figure S6). Another sensitivity analysis with similar SA methods was conducted excluding patients who have less than five years of continuous retrospective record, and it yielded similar clusters of the main analysis (Supplementary Figure S7).

## DISCUSSION

Our study examined health care patterns among 1,875 adult patients who sought consultation for MSK conditions in primary care settings and investigated the relationship between these health care patterns and the patients’ self‐reported MSK‐HQ outcomes. Using SA, we identified five distinct clusters that differed in terms of MSK‐related pain consultations, analgesic prescriptions, imaging, physiotherapy, and secondary care referrals. The data tell us that the low consultation and health care use group has the best MSK health. Factors associated with being in the other clusters and poorer health are sex, age, BMI, comorbidities, and neighborhood deprivation.

To our knowledge, this is the first study to use SA methodology to uncover health care patterns of MSK conditions in primary care using routinely collected EHR data. A Canadian study by Nguena Nguefack et al^6^ used SA to identify five two‐year care trajectories among patients living with arthritic conditions. However, their focus was on patterns of health care visits across different health care services (eg, emergency department visits, hospitalizations, and pain clinics) without considering multiple treatment strategies. This may be due to variations in the health care systems, which may influence the applicability of different primary care approaches. Similarly, the study by Mose et al^5^ employed latent class growth analysis to identify five 10‐year patterns of MSK health care utilization among adult Danes who reported chronic MSK pain. Although they modeled the number of health care contacts, they did not analyze the sequence of services used. Our findings have similarities with trajectories from studies analyzing single components of health care. However, in contrast, our study examined jointly all the main components of MSK management in primary care settings. Additionally, Meisingset et al^33^ identified five distinct MSK phenotypes using latent class analysis, but their focus was on key prognostic factors over the biopsychosocial domains across common MSK pain. Although these phenotypes may support the development of targeted interventions, our study, which integrates different care strategies for MSK pain in primary care, offers practical insights that may enhance clinical practice and inform decision‐making in primary care settings.

This study demonstrated that patients in the “high consultation and health care use” group experienced the worst outcome in terms of MSK‐HQ score. This finding aligns with the results of the study by Nguena Nguefack et al,^6^ which indicated that belonging to a high health care utilization group was associated with a higher likelihood of perceiving a poor or fair quality of life.^6^ This high‐utilization group in our study represented 8.32% of consulters with MSK pain and predominantly consisted of female participants, older patients, individuals with obesity, and those coming from the most deprived areas. Additionally, this group had the highest proportion of patients with a comorbidity count of three or above, suggesting a significant burden of comorbidities.^6^

In contrast, patients in the “low consultation and health care use” group exhibited the best MSK health (highest MSK‐HQ score). This was the largest group, comprising 57.39% of consulters with MSK pain, and included a higher proportion of male participants and younger patients, fewer individuals with obesity, and a greater proportion of patients with no comorbidities. Notably, 389 patients (36.15%) in this group had consultations only in year 1, suggesting they might be incident consulters. Furthermore, individuals from the least deprived areas typically use health care services less frequently than those from the most deprived areas, a finding consistent with other studies reporting socioeconomic differences in the prevalence and management of chronic pain.^34^ These results indicate that more sophisticated SA nevertheless confirms the general observation made in previous studies of a subset of patients with high levels of pain and disability and high health care use, in which issues of quality and effectiveness of care may be more important than simple lack of access to primary care.

By evaluating data from the five years before the index consultation, we gained insights into the longitudinal treatment strategies experienced by patients. This helps health care providers learn from previous cases, refining treatment guidelines and care strategies based on actual outcomes. Furthermore, our approach helps identify patient groups that require more intensive and tailored care, allowing for a more effective allocation of resources to where they are needed most. Our findings reveal that nearly half of the patients consulting for MSK conditions have a long history of health care interactions, which is associated with poorer short‐term outcome. These patients typically come from socioeconomically deprived areas, are predominantly older and female, and have obesity and multiple comorbidities. Our assessment of patients’ profiles and outcome variations among health care utilization patterns can be used to improve care pathways and highlights areas in which policy interventions could substantially enhance health equity.

The strength of this study lies in its innovative multidimensional approach to SA, enabling a comprehensive exploration of the most shared health care utilization patterns for MSK conditions in primary care, considering patterns of consultations, analgesic prescriptions, imaging, physiotherapy, and secondary care referrals. There are potential limitations in this study. The inclusion of only those patients who consented to participate might have introduced a selection bias, as evidenced by the poor response rate. Additionally, the IMD data suggest that the sample was less deprived compared to the general population. Consequently, the patterns of health care identified here, and their relative frequency, may not reflect those in the target population of all adult consulters with MSK pain. In particular, the frequency of low consultation and health care use may be overestimated in our sample, given indirect evidence of lower study participation among more deprived patients. Moreover, our analysis was based on continuous retrospective records of five years before the index consultation. The registration period of the patients was not available in the data, so we were not sure whether the patients with missing health care events had no recorded events or were not registered during that period. We checked whether the patients had five years of continuous records by computing the difference between the index date and the date of the first recorded event. We found 738 patients had less than five years of continuous retrospective record. Excluding these patients does reduce the sample size. Therefore, we conducted a sensitivity analysis excluding these patients, and the full results are provided in the supplementary file (Supplementary Figures S8–S11 and Supplementary Tables S4–S9).

Optimizing primary care and linkage to effective approaches is crucial for reducing the effect of MSK conditions. Understanding the patterns of patients’ journeys through various health care services contributes to the achievement of this goal. SA could serve as a feasible method for identifying patient interactions with the health care system by delineating sequences of care events and identifying distinct health care utilization patterns. This study offers initial insights into patterns of health care by consulters with MSK pain to primary care, which have been directed by clinicians. Further investigations are warranted to gain a deeper understanding of care patterns for MSK conditions in primary and secondary care settings and focus on specific MSK subpopulations such as OA and LBP.

In conclusion, this study identified five distinct health care patterns among adult patients with MSK conditions using SA. Patients’ self‐reported outcomes and sociodemographic profiles varied across the five clusters. Patients with high health care utilization reported poorer outcomes, whereas those with lower utilization had better outcomes. These findings underscore the association among socioeconomic status, extensive health care utilization, and poorer health outcome, emphasizing the need for targeted policy interventions to improve health equity and quality of care.

## AUTHOR CONTRIBUTIONS

All authors contributed to at least one of the following manuscript preparation roles: conceptualization AND/OR methodology, software, investigation, formal analysis, data curation, visualization, and validation AND drafting or reviewing/editing the final draft. As corresponding author, Dr Yu confirms that all authors have provided the final approval of the version to be published and takes responsibility for the affirmations regarding article submission (eg, not under consideration by another journal), the integrity of the data presented, and the statements regarding compliance with institutional review board/Declaration of Helsinki requirements.

## Supporting information


**Appendix S1:** Supplementary Information.

Diclosure form.
