# Supplementary material for: Sequence Analysis to Phenotype Health Care Patterns in Adults With Musculoskeletal Conditions Using Primary Care Electronic Health Records
Source: Arthritis Care Res (Hoboken). 2025 Apr 19;77(7):906–15. doi: 10.1002/acr.25514 (PMC12209494; doi:10.1002/acr.25514)
Supplement: Supplementary file 1 — Appendix S1: Supplementary Information. [file ACR-77-906-s002.docx]

**Supplementary materials**

**Supplementary Table S1. SNOMED CT code list for MSK conditions**

| **SNOMED CT CONCEPT ID** | **Preferred Term** |
| --- | --- |
| 1003721002 | Pain of joint of knee |
| 1003722009 | Pain of knee region |
| 10085004 | Metatarsalgia |
| 102556003 | Pain in upper limb |
| 102570003 | Inguinal pain |
| 103014001 | Cervical nerve root pain |
| 10380004 | Crushing injury of finger |
| 105606008 | Injury of musculoskeletal system |
| 10601006 | Pain in lower limb |
| 1088071000000108 | Pain in upper arm |
| 11049006 | Cervical radiculitis |
| 111222003 | Derangement of medial meniscus |
| 111243002 | Bursitis of knee |
| 111640008 | Closed fracture of radius |
| 1126007 | Knee locking |
| 11654001 | Achilles tendinitis |
| 118952005 | Joint finding |
| 122481008 | Hammer toe |
| 123536004 | Sprain of upper extremity |
| 12519004 | Sprain of sacroiliac ligament |
| 125594001 | Injury of shoulder region |
| 125596004 | Injury of elbow |
| 125597008 | Injury of forearm |
| 125598003 | Injury of wrist |
| 125599006 | Injury of hand |
| 125600009 | Injury of hip region |
| 125601008 | Injury of knee |
| 125602001 | Injury of lower leg |
| 125603006 | Injury of ankle |
| 125604000 | Injury of foot |
| 125605004 | Fracture of bone |
| 125606003 | Fracture of cervical spine |
| 125607007 | Fracture of thoracic spine |
| 125608002 | Fracture of lumbar spine |
| 12584003 | Bone pain |
| 12676007 | Fracture of radius |
| 127278005 | Injury of upper extremity |
| 127279002 | Injury of lower extremity |
| 129179000 | Piriformis syndrome |
| 134407002 | Chronic back pain |
| 135897006 | Salter-Harris type II |
| 13695006 | Fracture of pubic rami |
| 1539003 | Acquired trigger finger |
| 157265008 | Dislocation of hip joint |
| 157266009 | Dislocation of knee joint |
| 16114001 | Fracture of ankle |
| 161880003 | Stiff neck symptom |
| 161882006 | Stiff neck |
| 161891005 | Backache |
| 161892003 | Backache with radiation |
| 161894002 | Complaining of low back pain |
| 161896000 | Complaining of upper back ache |
| 162411004 | Complaining of stiffness |
| 162429009 | Symptom: upper limb |
| 16250001000004107 | Fracture of shoulder |
| 164539000 | O/E - joint movement painful |
| 17059001 | Prepatellar bursitis |
| 17222009 | Closed fracture of distal end of radius |
| 17883008 | Sprain of hip |
| 18171007 | Fracture of phalanx of finger |
| 18347007 | Spinal stenosis of lumbar region |
| 18876004 | Pain in finger |
| 19660004 | Disorder of soft tissue |
| 201819000 | Degenerative joint disease involving multiple joints |
| 201837004 | Localized primary osteoarthritis of the ankle and/or foot |
| 202031002 | Generalised arthritis |
| 202246002 | Recurrent dislocation of the patellofemoral joint |
| 202336002 | Acetabular labrum tear |
| 202381003 | Knee joint effusion |
| 202472008 | Hand joint pain |
| 202479004 | Acromioclavicular joint pain |
| 202480001 | Elbow joint pain |
| 202482009 | Wrist joint pain |
| 202487003 | Sacroiliac joint pain |
| 202490009 | Ankle joint pain |
| 202596000 | Clicking joint |
| 202606004 | Clicking hip |
| 202609006 | Clicking knee |
| 202664003 | Cervical myelopathy |
| 202683005 | Cervical spondylosis with radiculopathy |
| 202693003 | Lumbosacral spondylosis with radiculopathy |
| 202708005 | Prolapsed lumbar intervertebral disc |
| 202732003 | Disc prolapse with radiculopathy |
| 202733008 | Cervical disc prolapse with radiculopathy |
| 202735001 | Lumbar disc prolapse with radiculopathy |
| 202752002 | Lumbar discitis |
| 202757008 | Cervical disc disorder with radiculopathy |
| 202788007 | Degenerative lumbar spinal stenosis |
| 202794004 | Lumbago with sciatica |
| 202805003 | Sacroiliac disorder |
| 202841003 | Supraspinatus tendinitis |
| 202842005 | Partial thickness rotator cuff tear |
| 202843000 | Full thickness rotator cuff tear |
| 202849001 | Subacromial impingement |
| 202855006 | Lateral epicondylitis |
| 202856007 | Biceps tendinitis |
| 202863007 | Adductor tendinitis |
| 202881005 | Tibialis posterior tendinitis |
| 202882003 | Plantar fasciitis |
| 202888004 | Anterior shin splints |
| 202900007 | Synovitis and tenosynovitis |
| 202914007 | Extensor tenosynovitis of wrist |
| 202916009 | Extensor tenosynovitis of thumb |
| 202936005 | Ganglion and cyst of synovium, tendon and bursa |
| 202942009 | Ganglion of wrist |
| 203045001 | Dupuytren's disease of palm |
| 203082005 | Fibromyalgia |
| 203095000 | Spasm of back muscles |
| 203131005 | Tender heel pad |
| 203509009 | Clavicle pain |
| 203534009 | Acquired pes planus |
| 203601000 | Acquired unequal leg length |
| 203638000 | Kyphoscoliosis and scoliosis |
| 203639008 | Idiopathic scoliosis |
| 203645000 | Postural scoliosis |
| 203681002 | Acquired spondylolisthesis |
| 20511007 | Fracture of hand |
| 207940009 | Closed fracture thoracic vertebra wedge |
| 207957008 | Closed fracture lumbar vertebra |
| 207959006 | Closed fracture lumbar vertebra, wedge |
| 208145002 | Fracture or disruption of pelvis |
| 208165009 | Closed fracture pelvis single pubic ramus |
| 208166005 | Closed fracture pelvis multiple pubic rami - stable |
| 208240004 | Closed fracture proximal humerus, neck |
| 208242007 | Closed fracture proximal humerus, greater tuberosity |
| 208294009 | Closed fracture olecranon, extra-articular |
| 208325003 | Closed fracture radial styloid |
| 208388003 | Fracture at wrist and/or hand level |
| 208393000 | Fracture of metacarpal bone |
| 208394006 | Closed fracture of metacarpal bone |
| 208399001 | Closed fracture finger metacarpal neck |
| 208401007 | Closed fracture finger metacarpal |
| 208430000 | Closed fracture of one or more phalanges of hand |
| 208444006 | Closed fracture finger proximal phalanx |
| 208450001 | Closed fracture finger middle phalanx |
| 208634001 | Closed fracture distal tibia |
| 208687001 | Closed fracture metatarsal base |
| 208710000 | Closed fracture proximal phalanx, toe |
| 208712008 | Closed fracture distal phalanx, toe |
| 208719004 | Fracture of great toe |
| 2089002 | Paget's disease of bone |
| 208916003 | Acute meniscal tear medial posterior horn |
| 208921000 | Acute meniscal tear lateral |
| 209238002 | Closed fracture dislocation shoulder joint |
| 209354002 | Closed fracture dislocation foot |
| 209409002 | Sprains and strains of joints and adjacent muscles |
| 209436000 | Sprain of wrist and/or hand |
| 209520004 | Partial tear knee anterior cruciate ligament |
| 209529003 | Sprain of ankle and/or foot |
| 209532000 | Sprain, ankle joint, lateral |
| 209557005 | Neck sprain |
| 209565008 | Lumbar sprain |
| 209574005 | Pulled back muscle |
| 209629006 | Complete tear knee anterior cruciate ligament |
| 209812006 | Sprain, symphysis pubis |
| 21351003 | Fracture of phalanx of foot |
| 21698002 | Open fracture of phalanx of finger |
| 21794005 | Radial styloid tenosynovitis |
| 221695002 | Achilles bursitis |
| 22193007 | Degenerative joint disease of hand |
| 228158008 | Difficulty in walking |
| 22817005 | Strain of Achilles tendon |
| 22878006 | Contusion of knee |
| 23056005 | Sciatica |
| 23382007 | Stress fracture |
| 23406007 | Fracture of upper limb |
| 23482006 | Avulsion fracture |
| 235231000119100 | Osteophyte of bone |
| 23680005 | Enthesopathy |
| 239720000 | Tear of meniscus of knee |
| 239732001 | Disorder of patellofemoral joint |
| 239733006 | Anterior knee pain |
| 239863005 | Osteoarthritis of spinal facet joint |
| 239865003 | Osteoarthritis of acromioclavicular joint |
| 239866002 | Osteoarthritis of elbow |
| 239867006 | Osteoarthritis of wrist |
| 239868001 | Osteoarthritis of finger joint |
| 239872002 | Osteoarthritis of hip |
| 239873007 | Osteoarthritis of knee |
| 239874001 | Osteoarthritis of ankle |
| 239877008 | Osteoarthritis of first metatarsophalangeal joint |
| 239878003 | Osteoarthritis of toe joint |
| 239880009 | Lumbar spondylosis |
| 239960007 | Impingement syndrome of shoulder |
| 239961006 | Bursitis of shoulder |
| 240003004 | Suprapatellar bursitis |
| 240008008 | Synovial cyst of knee |
| 240131006 | Rhabdomyolysis |
| 240203005 | Rupture of Baker's cyst - knee |
| 240205003 | Synovial cyst |
| 240261009 | Generalised benign joint hypermobility |
| 240631000000102 | Suspected fracture or dislocation |
| 24424003 | Closed fracture of phalanx of finger |
| 24693007 | Myofascial pain syndrome |
| 247366003 | Acute back pain with sciatica |
| 247369005 | Facet joint pain |
| 247373008 | Ankle pain |
| 248491001 | Swollen knee |
| 250082003 | Hand cramps |
| 250102002 | Unstable knee |
| 25415003 | Closed fracture of femur |
| 25899002 | Closed bimalleolar fracture |
| 262520005 | Thumb injury |
| 262965006 | Strain of back muscle |
| 262971000 | Tendon injury - hand |
| 262981001 | Rupture of gastrocnemius tendon |
| 262992000 | Hamstring sprain |
| 263021005 | Anterior dislocation of shoulder joint |
| 263051004 | Subluxation of shoulder joint |
| 263054007 | Subluxation of finger |
| 263055008 | Subluxation of thumb |
| 263058005 | Subluxation of knee joint |
| 263084004 | Fracture dislocation of finger or thumb |
| 263128001 | Sprain of ligament of elbow |
| 263129009 | Sprain of ligament of finger |
| 263130004 | Sprain of ligament of thumb |
| 263133002 | Sprain of lateral ligament of ankle joint |
| 263199001 | Fracture of distal end of radius |
| 263225007 | Fracture of proximal end of femur |
| 263233008 | Closed fracture of femur, distal end |
| 263244000 | Bimalleolar fracture of ankle |
| 263246003 | Fracture of talus |
| 263247007 | Fracture of calcaneus |
| 263251009 | Metatarsal bone fracture |
| 26538006 | Degeneration of lumbar intervertebral disc |
| 267039000 | Swollen ankle |
| 267109007 | Symptom of ankle or foot |
| 267889007 | Generalised osteoarthritis of the hand |
| 267949000 | Shoulder joint pain |
| 267953003 | Arthralgia of the lower leg |
| 267954009 | Arthralgia of the ankle and/or foot |
| 267970006 | Cervical spondylosis without myelopathy |
| 267981009 | Pain in thoracic spine |
| 267982002 | Pain in lumbar spine |
| 269062008 | Closed fracture of cervical spine |
| 269080004 | Closed fracture of lower end of humerus |
| 269083002 | Closed Colles' fracture |
| 269105005 | Dislocation or subluxation of shoulder |
| 269111008 | Dislocation or subluxation of finger or thumb |
| 269112001 | Dislocation or subluxation of knee |
| 269113006 | Acute meniscal tear medial |
| 270476009 | Wry neck/torticollis |
| 270887007 | Rupture of popliteal space synovial cyst |
| 271587009 | Stiffness |
| 271771009 | Joint swelling |
| 27182002 | Sprain of acromioclavicular ligament |
| 272009001 | Complaining of a back symptom |
| 272014002 | Complaining of foot symptom |
| 2733002 | Heel pain |
| 274142002 | Dupuytren's contracture |
| 274160002 | Fracture of phalanx of thumb |
| 274162005 | Thoracic back sprain |
| 274179004 | Traumatic haematoma |
| 2764000 | Joint crepitus |
| 27741009 | Calcific tendinitis of shoulder |
| 277890004 | Swollen toe |
| 278860009 | Chronic low back pain |
| 278862001 | Acute low back pain |
| 279029001 | Pain in cervical spine |
| 279035001 | Acute thoracic back pain |
| 279038004 | Thoracic back pain |
| 279039007 | Low back pain |
| 279040009 | Mechanical low back pain |
| 279043006 | Pain in buttock |
| 279069000 | Musculoskeletal pain |
| 281531008 | Fracture of medial malleolus |
| 281535004 | Fracture of lateral malleolus |
| 281543009 | Strain of tendon of medial thigh muscle |
| 281598004 | Sprain of spinal ligament |
| 281792000 | Swollen lower leg |
| 281974002 | Weber type B fracture |
| 281975001 | Weber type A fracture |
| 282026002 | Soft tissue injury |
| 282766005 | Lower back injury |
| 282775007 | Calf injury |
| 282776008 | Injury of toe |
| 283858004 | Crush injury of thumb |
| 285365001 | Pain in toe |
| 285395009 | Strain of calf muscle |
| 29210001 | Trochanteric tendinitis |
| 297142003 | Swollen foot |
| 297193007 | Ganglion of hand |
| 297194001 | Ganglion of foot |
| 298382003 | Scoliosis deformity of spine |
| 298494008 | Scoliosis of thoracic spine |
| 298857005 | Shoulder joint painful on movement |
| 299037003 | Swollen hand |
| 299060006 | Swelling of finger |
| 299331007 | Bandy legged |
| 30085007 | Morton metatarsalgia |
| 300954003 | Pain in calf |
| 300955002 | Pain in thumb |
| 301813003 | Irritable hip |
| 302222008 | Elbow fracture - closed |
| 302941001 | Nonunion of fracture |
| 30556007 | Recurrent dislocation of shoulder region |
| 30760008 | Finger clubbing |
| 308153009 | Closed fracture of distal fibula |
| 309246000 | Osteoarthritis of foot joint |
| 309567004 | Toe problem |
| 30989003 | Knee pain |
| 310483003 | Complaining of pain in toe |
| 310484009 | Complaining of pain in hallux |
| 311804006 | Prolapsed lumbar intervertebral disc with sciatica |
| 311821002 | Closed fracture of great toe |
| 312225001 | Musculoskeletal and connective tissue diseases |
| 314916002 | Swollen thumb |
| 31975004 | Fracture of navicular bone of wrist |
| 31978002 | Fracture of tibia |
| 3199001 | Sprain of shoulder |
| 33173003 | Closed fracture of clavicle |
| 33192001 | Closed fracture of lower end of radius AND ulna |
| 33308003 | Disorder of back |
| 342070009 | Closed fracture of foot |
| 34268009 | Closed fracture of lateral malleolus |
| 34789001 | Pain in the coccyx |
| 34840004 | Tendinitis |
| 35678005 | Multiple joint pain |
| 359532006 | Rotator cuff impingement syndrome |
| 359817006 | Closed fracture of hip |
| 359820003 | Closed fracture of neck of femur |
| 360450007 | Strain of neck muscle |
| 36071006 | Chondromalacia of patella |
| 36186002 | Polyarthropathy |
| 36427004 | Intervertebral disc disorder |
| 36924003 | Closed fracture of metatarsal bone |
| 371081002 | Arthritis of knee |
| 371598009 | Heberden node |
| 37785001 | Patellar tendonitis |
| 37895003 | Osteoarthrosis of the carpometacarpal joint of the thumb |
| 387800004 | Cervical spondylosis |
| 387802007 | Thoracic spondylosis |
| 396275006 | Osteoarthritis |
| 39848009 | Whiplash injury to neck |
| 398878007 | Sprain of ligament |
| 399114005 | Adhesive capsulitis of shoulder |
| 399269003 | Arthropathy |
| 399346004 | Supraspinatus tear |
| 40144003 | Morning stiffness - joint |
| 404098005 | Digital mucous cyst |
| 4046000 | Degenerative spondylolisthesis |
| 405817008 | Fracture of phalanx of hand |
| 40799003 | Subacromial bursitis |
| 4106009 | Rotator cuff syndrome |
| 413428007 | Acquired kyphosis |
| 413875004 | Closed fracture of head of humerus |
| 414293001 | Fracture of tibia AND fibula |
| 41511005 | Open fracture of distal phalanx of finger |
| 415692008 | Swelling of first metatarsophalangeal joint of hallux |
| 416189003 | Exostosis |
| 416209007 | Synovitis |
| 417076003 | Dislocation of shoulder joint |
| 417109008 | Subluxation of radial head |
| 417163006 | Traumatic AND/OR non-traumatic injury |
| 417558002 | Dislocation of elbow joint |
| 417746004 | Traumatic injury |
| 418237007 | Pain in hallux |
| 42188001 | Closed fracture of ankle |
| 423849004 | Iliotibial band friction syndrome |
| 424648000 | Closed fracture of base of fifth metatarsal bone |
| 425940002 | Olecranon bursitis |
| 42636007 | Closed fracture of upper end of humerus |
| 42786005 | Snapping thumb syndrome |
| 428151000 | Closed fracture of bone of knee joint |
| 42818005 | Closed fracture of scaphoid bone of wrist |
| 428257007 | Fracture of tibial plateau |
| 428883008 | Rupture biceps tendon |
| 429513001 | Rupture Achilles tendon |
| 4308002 | Repetitive strain injury |
| 432473000 | Femoral acetabular impingement |
| 43295006 | Closed fracture of humerus |
| 43422002 | Crushing injury of foot |
| 442048005 | Tenosynovitis of wrist |
| 442056008 | Torus fracture |
| 442085002 | Greenstick fracture |
| 442520000 | Inflammation of rotator cuff tendon |
| 443700006 | Disorder of lumbar disc |
| 443798008 | Arthritis of shoulder region joint |
| 44465007 | Sprain of ankle |
| 445008009 | Ganglion |
| 447139008 | Closed fracture of tibia |
| 447395005 | Closed fracture of fibula |
| 448355005 | Greenstick fracture of distal radius |
| 448394006 | Inflammation of joint of foot |
| 448589005 | Arthritis of hand |
| 449917004 | Cramp in lower limb |
| 450521003 | Osteoarthritis of patellofemoral joint |
| 45231001 | Infrapatellar bursitis |
| 45326000 | Shoulder pain |
| 45352006 | Spasm of muscle |
| 45613006 | Contusion of lower leg |
| 46866001 | Fracture of lower limb |
| 47933007 | Foot pain |
| 481000119104 | Strain of hamstring muscle |
| 48210000 | Lumbosacral spondylosis without myelopathy |
| 48532005 | Muscle strain |
| 49218002 | Hip pain |
| 49388007 | Sprain of foot |
| 51037009 | Fracture of patella |
| 52011008 | Injury of finger |
| 53057004 | Hand pain |
| 53208009 | Peroneal tendinitis |
| 53226007 | Pes planus |
| 53286005 | Medial epicondylitis of elbow joint |
| 53627009 | Closed fracture of radius AND ulna |
| 54556006 | Fracture of ulna |
| 54888009 | Sprain of knee |
| 55146009 | Sacroiliitis |
| 55260003 | Calcaneal spur |
| 55300003 | Cramp |
| 56608008 | Pain in wrist |
| 57406009 | Carpal tunnel syndrome |
| 57676002 | Joint pain |
| 58150001 | Fracture of clavicle |
| 58580000 | Closed supracondylar fracture of humerus |
| 58781003 | Gluteal tendinitis |
| 5913000 | Fracture of neck of femur |
| 61007003 | Separation of symphysis pubis during delivery |
| 62629000 | Divarication of recti |
| 637091000000105 | Osteoarthritis NOS, of the lower leg |
| 64217002 | Curvature of spine |
| 64298006 | Mallet finger |
| 64455005 | Fracture of acetabulum |
| 64665009 | Closed fracture of calcaneus |
| 65260001 | Cervical spondylosis with myelopathy |
| 65358001 | Acquired hallux valgus |
| 65966004 | Fracture of forearm |
| 66308002 | Fracture of humerus |
| 6654000 | Acquired hallux rigidus |
| 6698000 | Closed trimalleolar fracture |
| 67315001 | Degenerative joint disease of shoulder region |
| 67801009 | Tenosynovitis |
| 68449006 | Arthritis of hip |
| 6858004 | Capsulitis |
| 68854005 | Closed fracture of head of radius |
| 68962001 | Muscle pain |
| 69195002 | Degeneration of cervical intervertebral disc |
| 699062006 | Injury of shoulder and upper arm |
| 699370008 | Symptom of foot |
| 699462004 | Monoarthritis |
| 70070008 | Torticollis |
| 704213001 | Closed fracture of phalanx of thumb |
| 70704007 | Sprain of wrist |
| 712893003 | Traumatic and/or non-traumatic injury of back |
| 71620000 | Fracture of femur |
| 72047008 | Osgood Schlatter disease |
| 721291009 | Disorder of patella, unspecified |
| 73589001 | Intervertebral disc prolapse |
| 74323005 | Pain in elbow |
| 74779009 | Strain of rotator cuff capsule |
| 74814004 | Contusion of foot |
| 75308009 | Closed fracture of navicular bone of foot |
| 75591007 | Fracture of fibula |
| 75857000 | Fracture of radius AND ulna |
| 76107001 | Spinal stenosis |
| 7674000 | Greater trochanteric pain syndrome |
| 76865005 | Closed fracture of distal phalanx of finger |
| 771083005 | Pain in upper arm |
| 77493009 | Fracture of pelvis |
| 77547008 | Degeneration of intervertebral disc |
| 78435003 | Ganglion of joint |
| 78514002 | Thigh pain |
| 788465007 | Repetitive motion disorder |
| 789758005 | Strain of rotator cuff of shoulder |
| 80068009 | Swelling of limb |
| 80692000 | Late effect of tendon injury |
| 80756009 | Closed fracture of patella |
| 81498004 | Bursitis of hip |
| 81576005 | Closed fracture of phalanx of foot |
| 81680005 | Neck pain |
| 81902001 | Sprain of medial collateral ligament of knee |
| 82423001 | Chronic pain |
| 82675004 | Baker's cyst |
| 82991003 | Generalized aches and pains |
| 83561009 | Spinal stenosis in cervical region |
| 84017003 | Bursitis |
| 84445001 | Joint stiffness |
| 84869007 | Musculoskeletal symptoms |
| 85551004 | Hypermobility syndrome |
| 86380000 | Acquired claw toes |
| 870206000 | Weber type A fracture |
| 870207009 | Weber type B fracture |
| 87778004 | Sprain of hand |
| 8847002 | Spondylosis |
| 88998003 | Osteophyte |
| 90460009 | Injury of neck |
| 906591000006102 | Soft tissue injuries |
| 90834002 | Pain in limb |
| 91037003 | Closed fracture of pelvis |
| 926335004 | Rupture of rotator cuff of shoulder |
| 928000 | Disorder of musculoskeletal system |
| 939761000006103 | Musculoskeletal pain mild |
| 9468002 | Closed fracture of carpal bone |
| 95414005 | Calcific tendinitis |
| 95854004 | Pulled elbow |
| 9682006 | Fracture of scapula |
| 9808005 | Closed fracture of cuboid bone of foot |
| 1931871000006102 | Suspected fracture |

**Supplementary Appendix S1.** **Defining categorical states**

In year 1, all patients had at least one consultation. In years 2-5, 50-60% of patients had no consultations, 25-35% had 1–3 consultations per year, and the remaining patients had 4 or more consultations. Therefore, we defined three categorical states for consultations: “None”, “Low”, and “High” representing 0, 1–3, and 4 or more instances, respectively. The same categorisation was used for prescriptions. For imaging and physiotherapy referrals, patients were categorised as “No” and “Yes” based on whether they had no referrals or at least one referral during the year. Similarly, for secondary care referrals, patients were categorised as “No” and “Yes” based on whether they had no referrals or at least one referral in the MSK triage, rheumatology, or trauma and orthopedics department during the year.

**Supplementary Appendix S2. Selection of optimum number of clusters**

The decision of optimum number of clusters was based on the dendrogram, and inertia jump curve, cluster quality indices, and clinical relevance and interpretability. A dendrogram in cluster analysis is a tree-like diagram that illustrates how data points or clusters are grouped based on their similarity or dissimilarities [1]. Inertia, also known as within-cluster sum of squares, measures the dispersion of data points within clusters. Analysing the inertia curve helps to determine the optimal number of clusters by identifying the point where the rate of decrease in inertia slows down significantly, often referred to as the "elbow point" [2]. Different indices can be used to measure the quality of clusters [3]. Supplementary Table S2 presents the best number of groups according to each quality measure and the value of these statistics. Maximum values indicate better cluster quality for all indices except for the HC index. According to the PBC measure, partitioning into five groups is the best solution, whereas the ASW index suggests that a solution with two groups is preferable. Supplementary Figure S3 shows that a solution with five groups is a local maximum for the ASW, CH, HC, and PBC measures.

**Supplementary Figure S1. Flow chart of sample selection process**

**
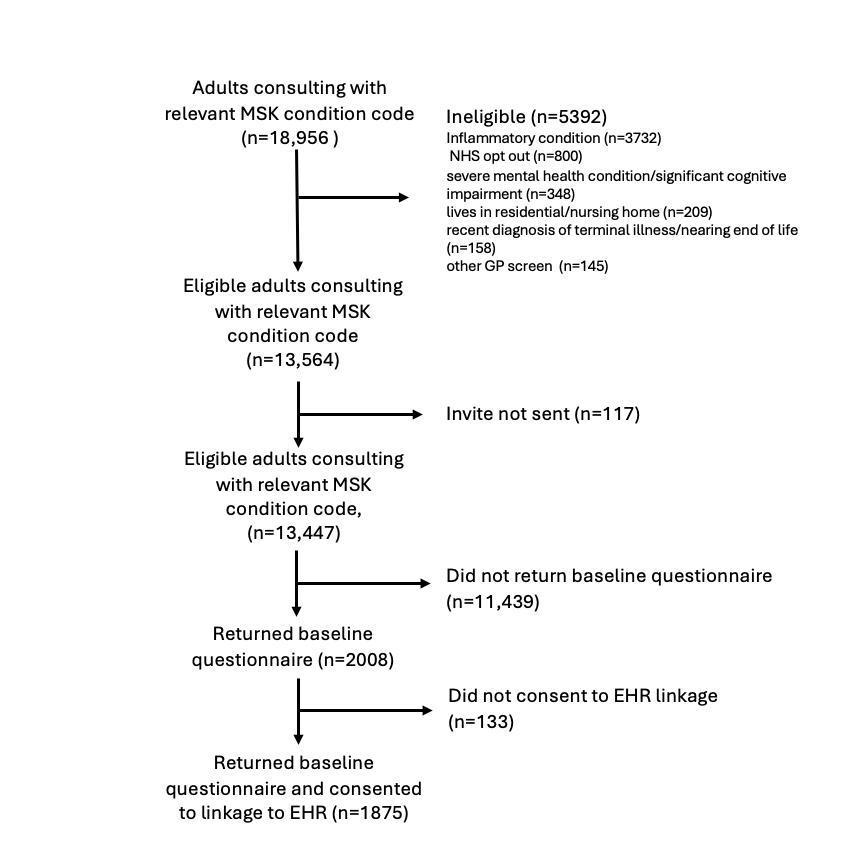
**

**Supplementary Figure S2. Sequence index plot of care sequence by domain (consultations, prescriptions, imaging, physiotherapy, and secondary care referrals). In sequence index plots, each line represents an individual’s care sequence.**


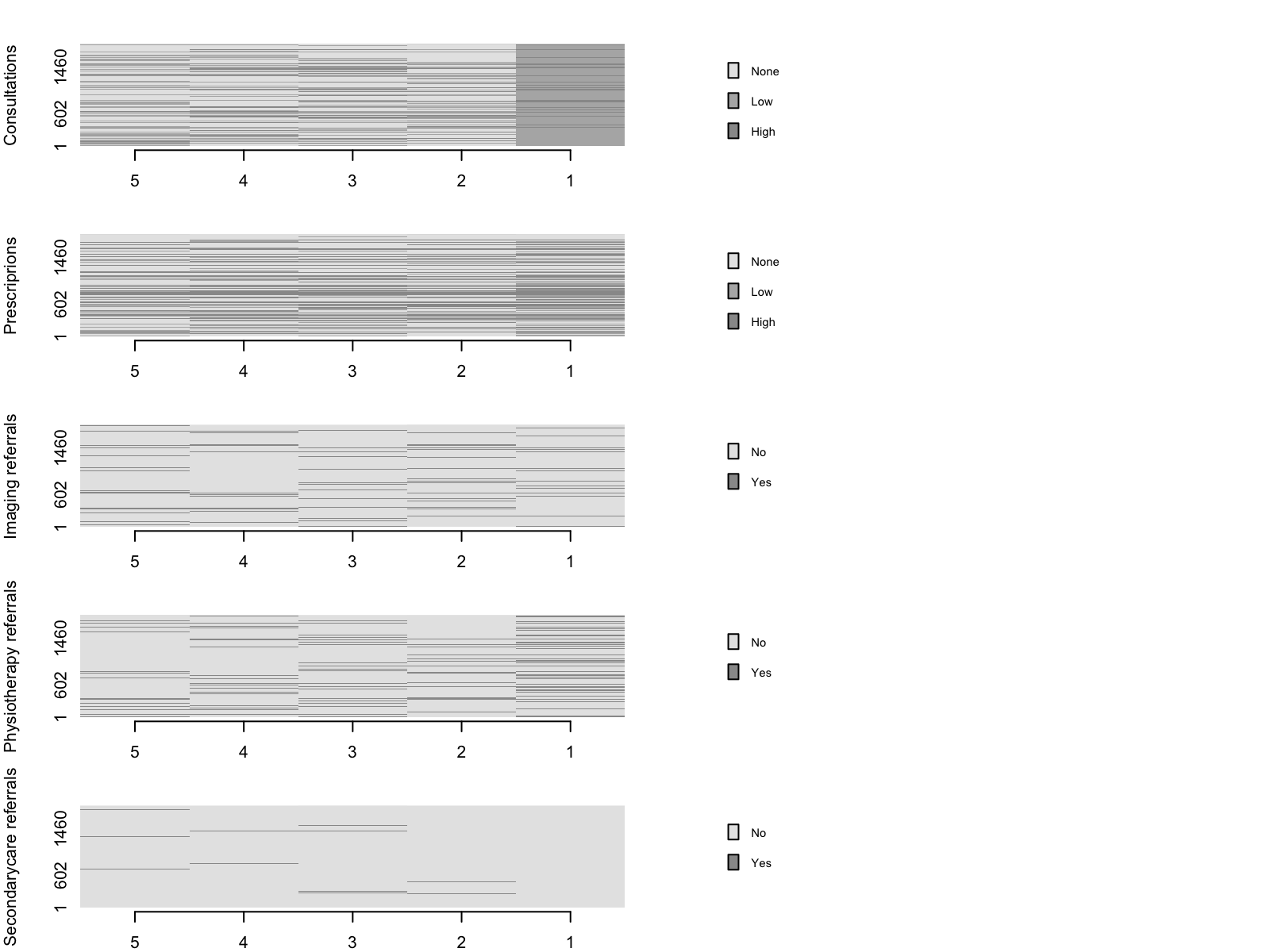


**Supplementary Table S2. Measures of quality of clusters**

| Indices | Groups | Statistics |
| --- | --- | --- |
| PBC | 5 | 0.6564 |
| HG | 5 | 0.7940 |
| HGSD | 5 | 0.7940 |
| ASW | 2 | 0.3365 |
| ASWw | 2 | 0.3372 |
| CH | 2 | 315.65 |
| R2 | 10 | 0.3135 |
| CHsq | 2 | 666.34 |
| R2sq | 10 | 0.4911 |
| HC | 5 | 0.0925 |

**Supplementary Figure S3. Cluster quality indices according to the number of clusters**


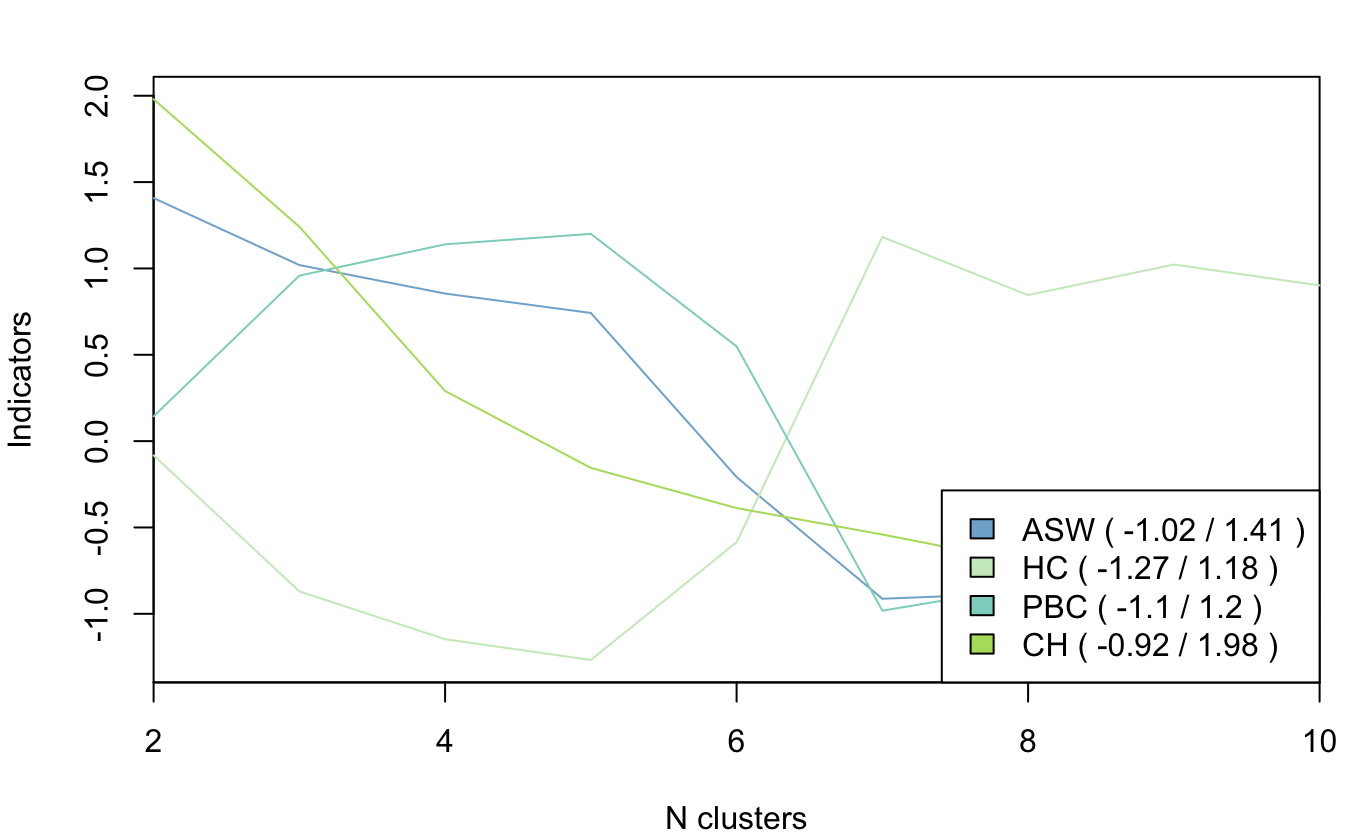


**Supplementary Figure S4. Hierarchical cluster analysis – dendrogram and inertia jump curve**

| 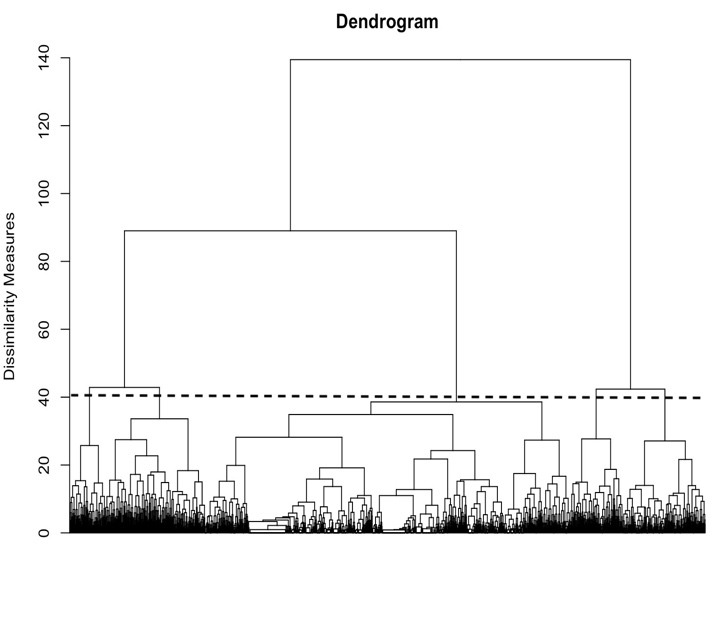 | 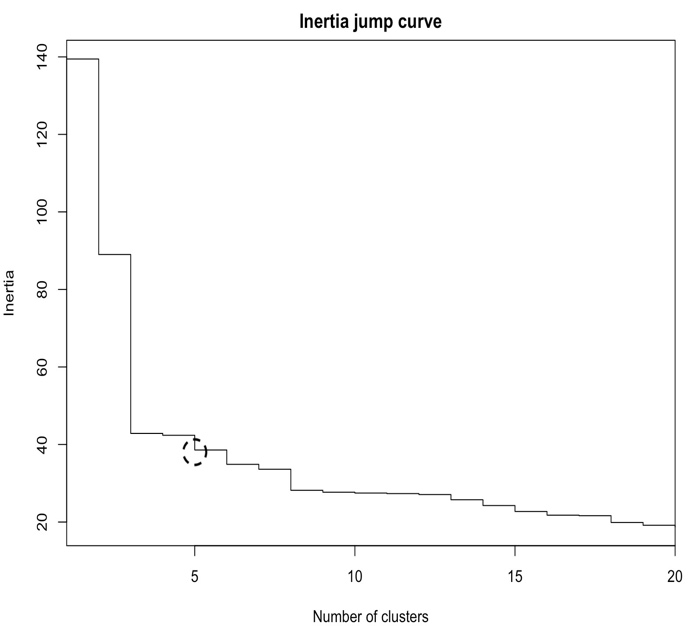 |
| --- | --- |

**Supplementary Table S3. Patients’ demographic and health characteristics by care sequence typology**

|  | Increasing consultation and analgesia | Low consultation and healthcare use | High consultation and healthcare use | Low consultation but high analgesia | Low consultation but moderate healthcare use | P- Value |
| --- | --- | --- | --- | --- | --- | --- |
|  | n= 105 (5.60%) | n= 1076 (57.39%) | n= 156 (8.32%) | n= 244 (13.01%) | n= 294 (15.68%) |  |
| Gender, n (%) | |  |  |  |  | **<0.001** |
| Male | 33 (31.43) | 420 (39.03) | 36 (23.08) | 74 (30.33) | 79 (26.87) |  |
| Female | 72 (68.57) | 656 (60.97) | 120 (76.92) | 170 (69.677) | 215 (73.13) |  |
| Age, Mean (SD) | 58.04 (15.92) | 55.14 (15.58) | 62.09 (14.34) | 64.30 (13.44) | 59.41 (14.98) | **<0.001** |
| Age-group, n (%) | |  |  |  |  | **<0.001** |
| 18-34 years | 9 (8.57) | 120 (11.15) | 6 (3.85) | 4 (1.64) | 18 (6.12) |  |
| 35-44 years | 8 (7.62) | 163 (15.15) | 11 (7.05) | 17 (6.97) | 35 (11.90) |  |
| 45-54 years | 26 (24.76) | 212 (19.70) | 29 (18.59) | 40 (16.39) | 55 (18.71) |  |
| 55-64 years | 20 (19.05) | 255 (23.70) | 36 (23.08) | 54 (22.13) | 65 (22.11) |  |
| 65-74 years | 24 (22.86) | 205 (19.05) | 39 (25.00) | 67 (27.46) | 72 (24.49) |  |
| 75-84 years | 16 (15.24) | 103 (9.57) | 29 (18.59) | 54 (22.13) | 39 (13.27) |  |
| 85+ years | 2 (1.90) | 18 (1.67) | 6 (3.85) | 8 (3.28) | 10 (3.40) |  |
| BMI, n (%) |  |  |  |  |  | **<0.001** |
| Underweight/Normal (<25) | 19 (20.88) | 280 (30.87) | 24 (17.91) | 45 (21.43) | 57 (22.53) |  |
| Overweight (25-29.9) | 24 (26.37) | 347 (38.26) | 32 (23.88) | 72 (34.29) | 87 (34.39) |  |
| Obese (≥30) | 48 (52.75) | 280 (30.87) | 78 (58.21) | 93 (44.29) | 109 (43.08) |  |
| Comorbidity count | |  |  |  |  | **<0.001** |
| 0 | 36 (34.29) | 570 (52.97) | 38 (24.36) | 60 (24.59) | 125 (42.52) |  |
| 1 | 37 (35.24) | 322 (29.93) | 38 (24.36) | 80 (32.79) | 100 (34.01) |  |
| 2 | 20 (19.05) | 142 (13.20) | 52 (33.33) | 70 (28.69) | 45 (15.31) |  |
| 3+ | 12 (11.43) | 42 (3.90) | 28 (17.95) | 34 (13.93) | 24 (8.16) |  |
| Index of Multiple Deprivation, n (%) | | |  |  |  | 0.053 |
| Quintile 1 (most deprived) | 34 (32.38) | 287 (26.67) | 56 (35.90) | 70 (28.69) | 83 (28.23) |  |
| Quintile 2 | 24 (22.86) | 202 (18.77) | 42 (26.92) | 54 (22.13) | 61 (20.75) |  |
| Quintile 3 | 17 (16.19) | 246 (22.86) | 19 (12.18) | 54 (22.13) | 62 (21.09) |  |
| Quintile 4 | 16 (15.24) | 189 (17.57) | 26 (16.67) | 43 (17.62) | 46 (15.65) |  |
| Quintile 5 (least deprived) | 14 (13.33) | 152 (14.13) | 13 (8.33) | 23 (9.43) | 42 (14.29) |  |

**Supplementary Figure S5. Sensitivity analysis: State distribution plot of care sequence typology by domain (consultations, analgesic prescriptions, imaging, physiotherapy, and secondary care referrals) for osteoarthritis patients**


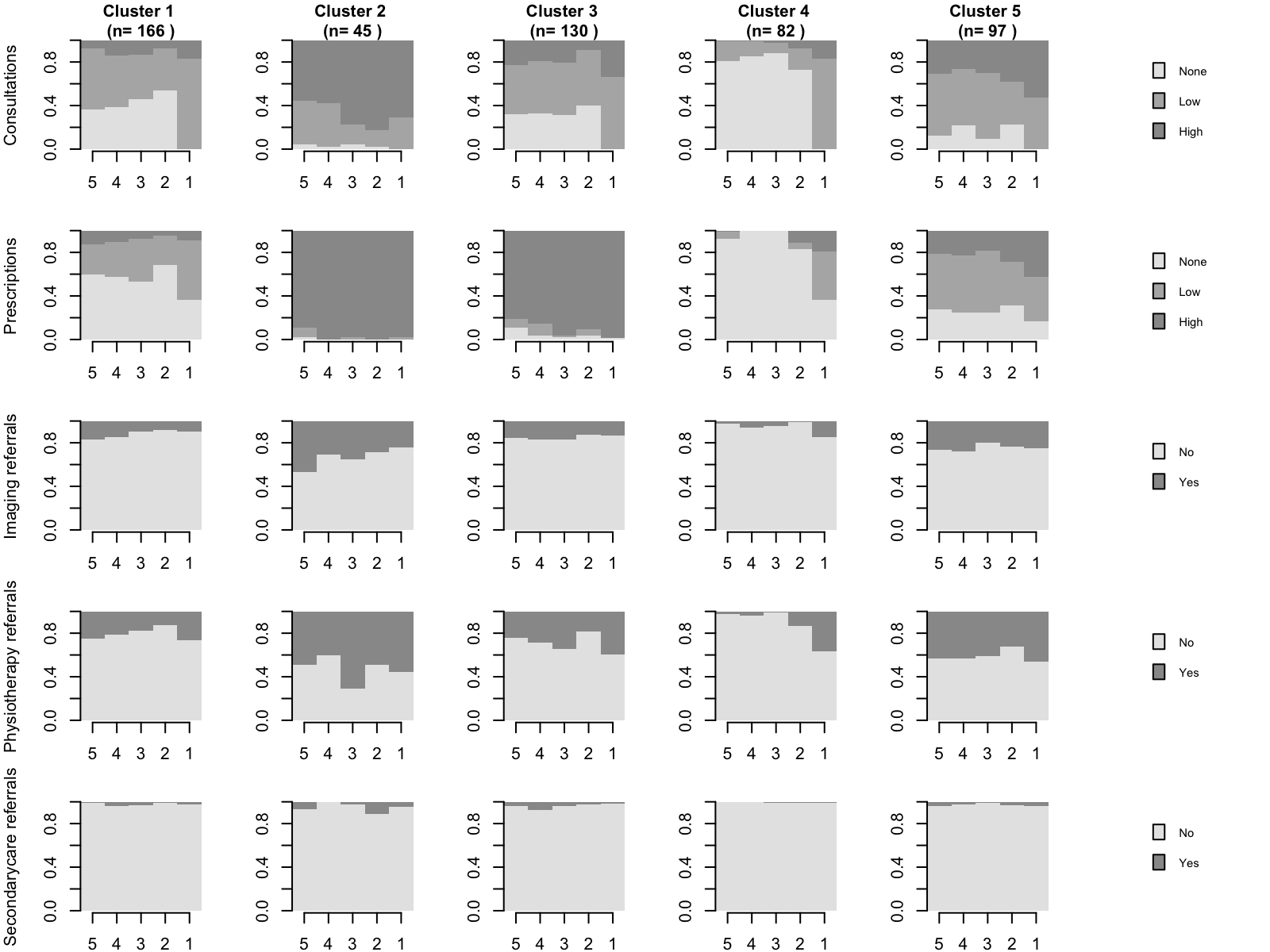


**Supplementary Figure S6. Sensitivity analysis: State distribution plot of care sequence typology by domain (consultations, analgesic prescriptions, imaging, physiotherapy, and secondary care referrals) for low back pain patients**


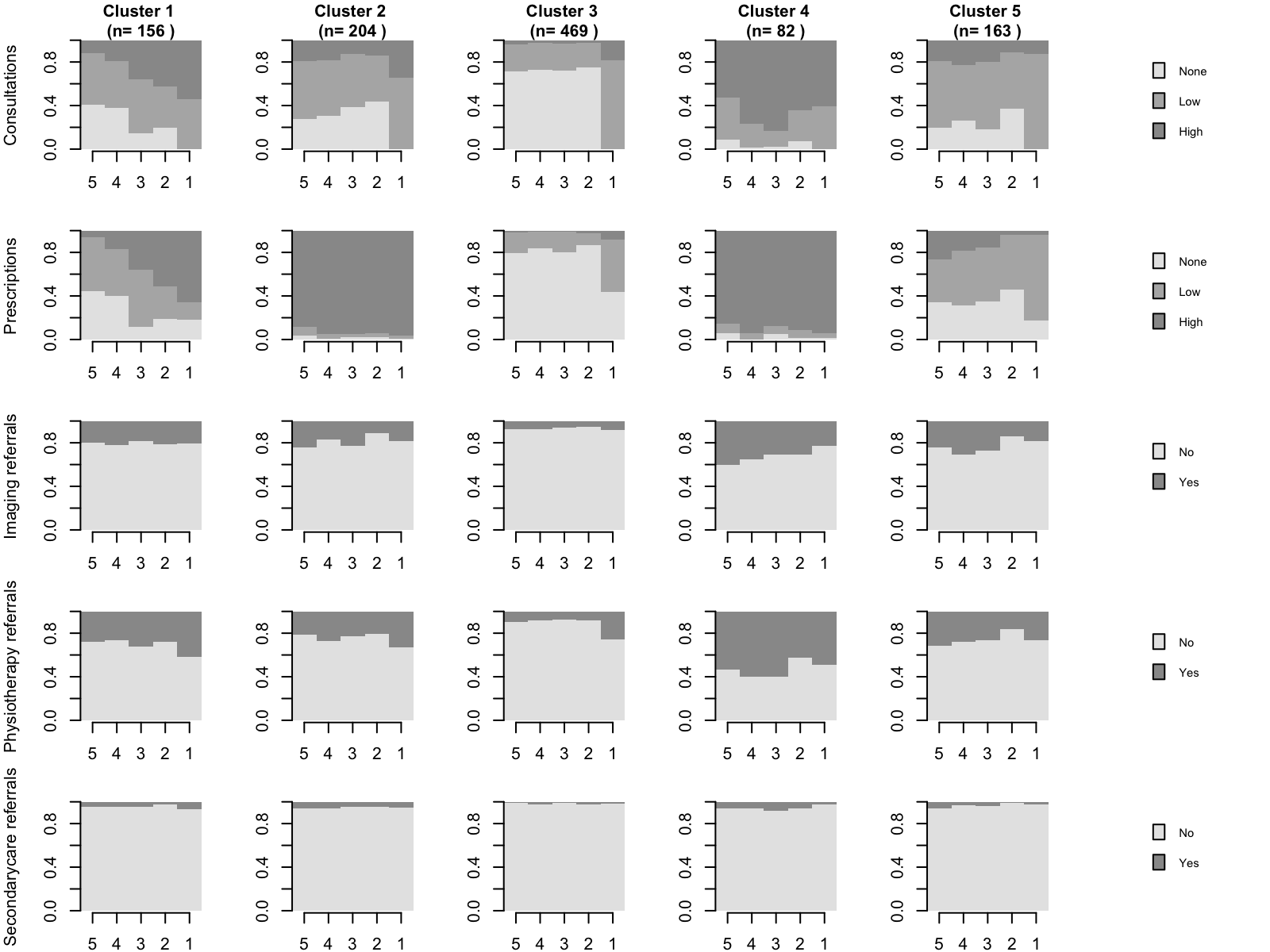


**Supplementary Figure S7. State distribution plot of care sequence typology by domain (consultations, analgesic prescriptions, imaging, physiotherapy, and secondary care referrals) for MSK patients with 5 years of continuous retrospective record**


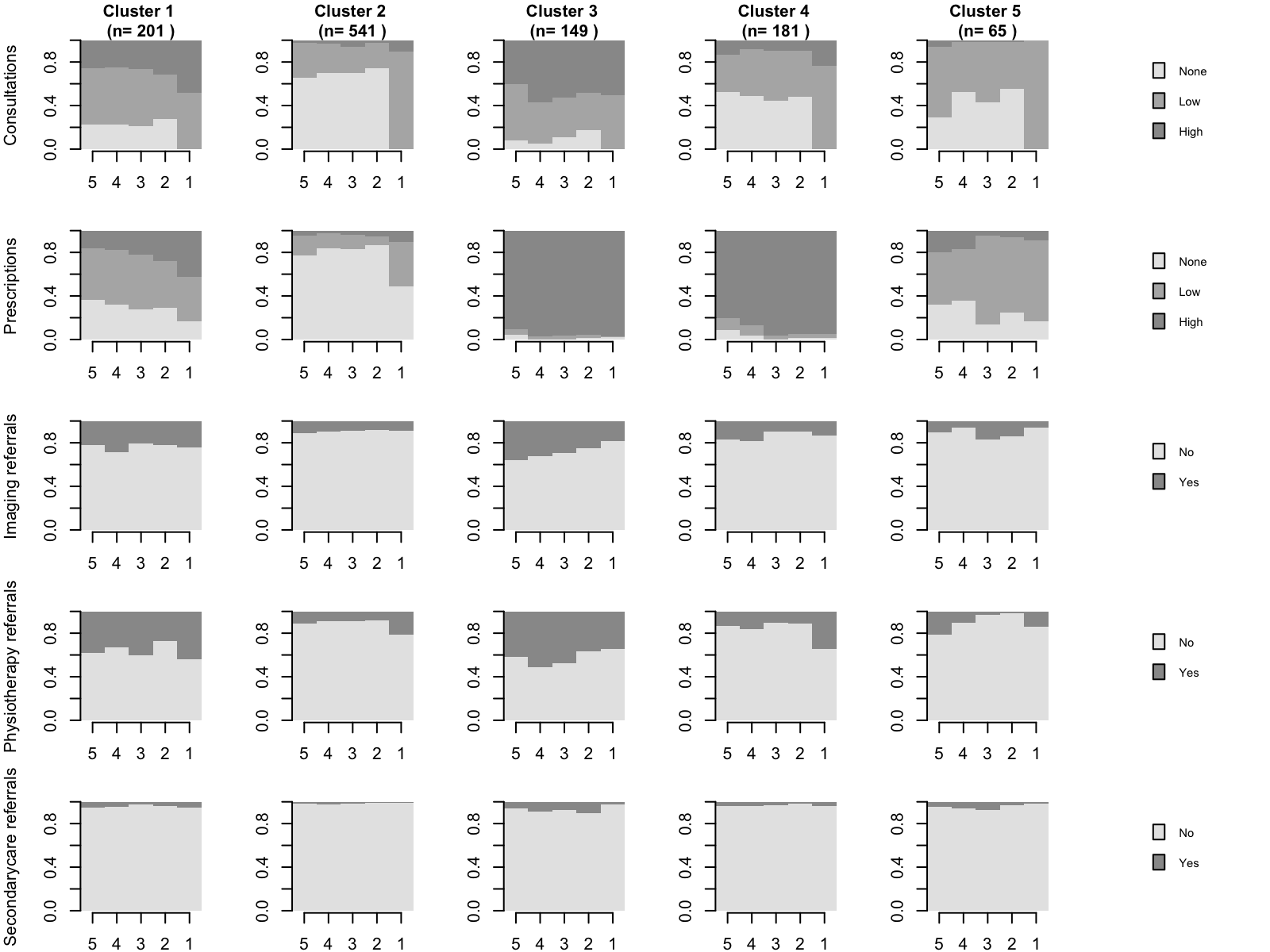


**Sensitivity analysis: Excluding patients with <5 years of continuous retrospective records (n=1137)**

**Supplementary Figure S8. Hierarchical cluster analysis – dendrogram and inertia jump curve**

| 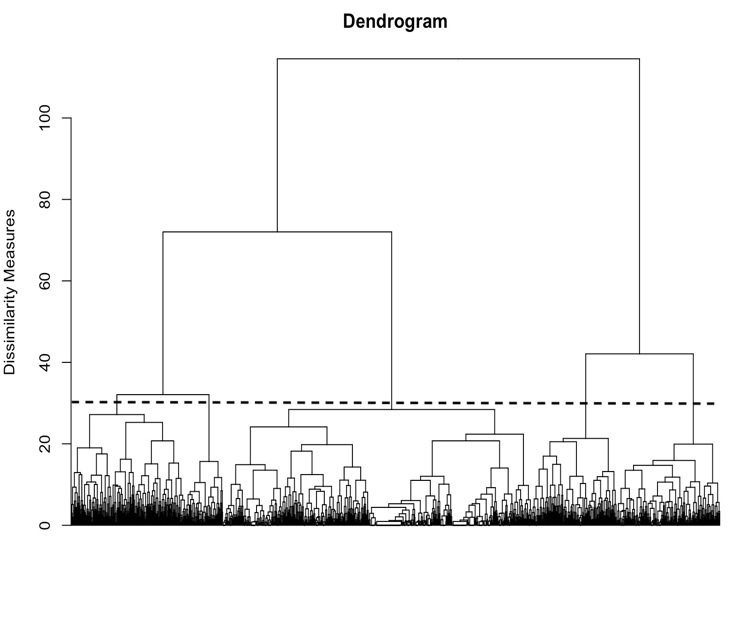 | 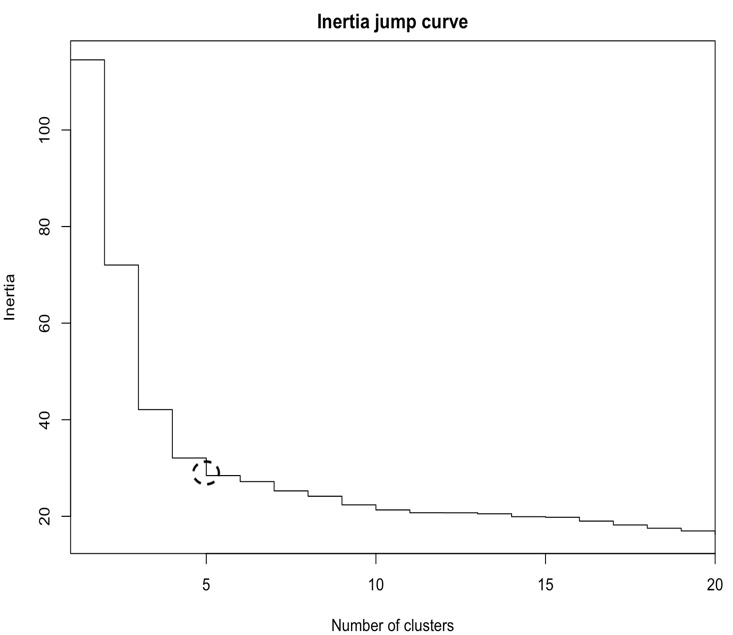 |
| --- | --- |

**Supplementary Figure S9. Cluster quality indices according to the number of clusters**


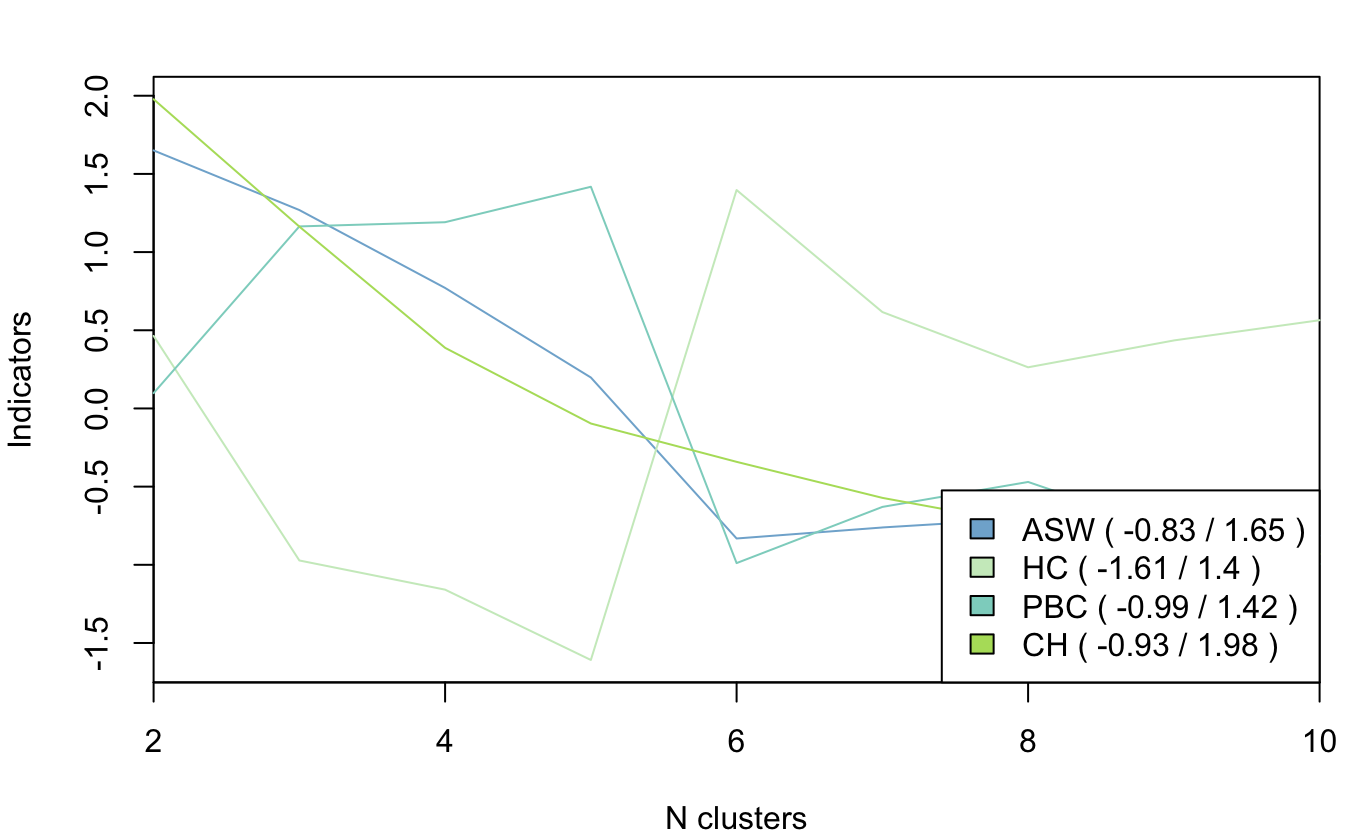


**Supplementary Figure S10. State distribution plot of care sequence typology by domain (consultations, analgesic prescriptions, imaging, physiotherapy, and secondary care referrals) for MSK patients**


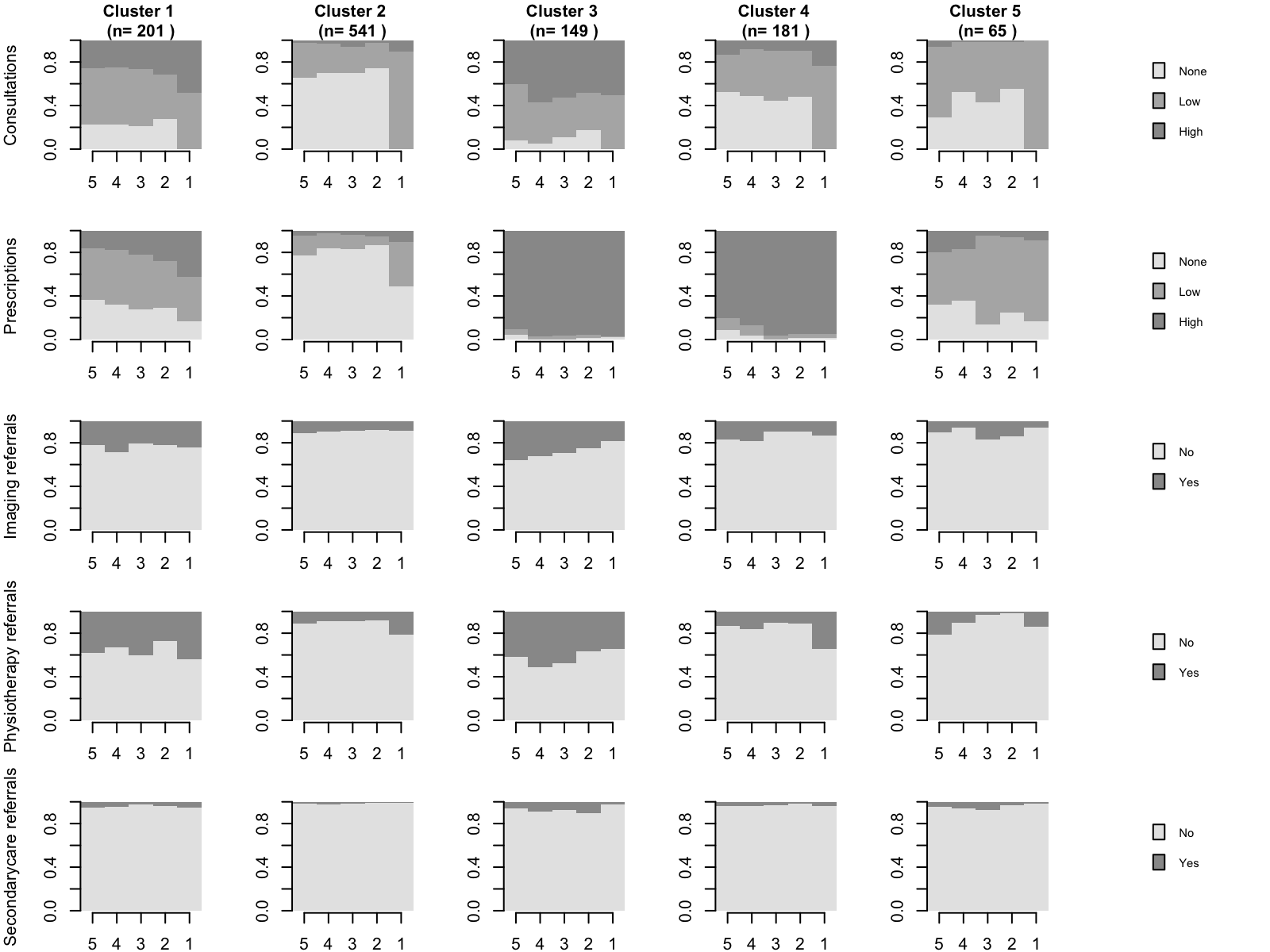


**Supplementary Figure S11. Predicted values of MSK-HQ score between the distinct clusters. Predicted values were controlled for gender, age, BMI, comorbidity count, and index of multiple deprivation.**

**Supplementary Table S4. Measures of quality of clusters**

| Indices | Groups | Statistics |
| --- | --- | --- |
| PBC | 5 | 0.5932 |
| HG | 5 | 0.7394 |
| HGSD | 5 | 0.7394 |
| ASW | 2 | 0.2886 |
| ASWw | 2 | 0.2899 |
| CH | 2 | 196.35 |
| R2 | 10 | 0.3101 |
| CHsq | 2 | 407.10 |
| R2sq | 10 | 0.4906 |
| HC | 5 | 0.1192 |

**Supplementary Table S5. Patients’ baseline characteristics**

| Variables (n=1137) | |
| --- | --- |
| Gender, n (%) | |
| Female | 794 (69.83) |
| Male | 343 (30.17) |
| Age, Mean (SD) | 60.23 (14.89) |
| Age-group, n (%) | |
| 18-34 years | 61 (5.36) |
| 35-44 years | 124 (10.91) |
| 45-54 years | 206 (18.12) |
| 55-64 years | 256 (22.52) |
| 65-74 years | 278 (24.45) |
| 75-84 years | 176 (15.48) |
| 85+ years | 36 (3.17) |
| BMI, Mean (SD) | 29.55 (6.75) |
| BMI*, n (%) |  |
| Underweight (<18.5) | 17 (1.50) |
| Normal (18.5-24.9) | 228 (20.05) |
| Overweight (25-29.9) | 330 (29.02) |
| Obese (≥30) | 395 (34.74) |
| Missing | 167 (14.69) |
| Comorbidity count, n (%) | |
| 0 | 422 (37.12) |
| 1 | 373 (32.81) |
| 2 | 230 (20.23) |
| 3+ | 112 (9.85) |
| Index of Multiple Deprivation, n (%) | |
| Quintile 1 (most deprived) | 328 (28.85) |
| Quintile 2 | 240 (21.11) |
| Quintile 3 | 234 (20.58) |
| Quintile 4 | 189 (16.62) |
| Quintile 5 (least deprived) | 146 (12.84) |
| Ethnicity, n (%) | |
| White | 1094 (96.22) |
| Asian | 19 (1.67) |
| Mixed | 4 (0.35) |
| Black | 14 (1.23) |
| Other | 6 (0.53) |

**Supplementary Table S6. Patients’ demographic and health characteristics by care sequence typology**

|  | Increasing consultation and analgesia | Low consultation and healthcare use | High consultation and healthcare use | Low consultation but high analgesia | Low consultation but moderate healthcare use | P- Value |
| --- | --- | --- | --- | --- | --- | --- |
|  | n= 201 (17.68%) | n= 541 (47.58%) | n= 149 (13.10%) | n=181 (15.92%) | n= 65 (5.72%) |  |
| Gender, n (%) | |  |  |  |  | 0.003 |
| Male | 59 (29.35) | 192 (35.49) | 33 (22.15) | 43 (23.76) | 16 (24.62) |  |
| Female | 142 (70.65) | 349 (64.51) | 116 (77.85) | 138 (76.24) | 49 (75.38) |  |
| Age, Mean (SD) | 59.16 (14.47) | 57.80 (15.15) | 62.06 (13.29) | 66.63 (13.50) | 61.58 (15.62) | <0.001 |
| Age-group, n (%) | |  |  |  |  | <0.001 |
| 18-34 years | 12 (5.97) | 41 (7.58) | 4 (2.68) | 2 (1.10) | 2 (3.08) |  |
| 35-44 years | 19 (9.45) | 75 (13.86) | 10 (6.71) | 10 (5.52) | 10 (15.38) |  |
| 45-54 years | 44 (21.89) | 101 (18.67) | 31 (20.81) | 21 (11.60) | 9 (13.85) |  |
| 55-64 years | 46 (22.89) | 125 (23.11) | 34 (22.82) | 39 (21.55) | 12 (18.46) |  |
| 65-74 years | 51 (25.37) | 120 (22.18) | 43 (28.86) | 48 (26.52) | 16 (24.62) |  |
| 75-84 years | 24 (11.94) | 66 (12.20) | 22 (14.77) | 51 (28.18) | 13 (20.00) |  |
| 85+ years | 5 (2.49) | 13 (2.40) | 5 (3.36) | 10 (5.52) | 3 (4.62) |  |
| BMI, n (%) |  |  |  |  |  | <0.001 |
| Underweight/Normal (<25) | 31 (18.45) | 133 (29.04) | 25 (19.38) | 44 (27.85) | 12 (21.05) |  |
| Overweight (25-29.9) | 49 (29.17) | 171 (37.34) | 30 (23.26) | 55 (34.81) | 25 (43.86) |  |
| Obese (≥30) | 88 (52.38) | 154 (33.62) | 74 (57.36) | 59 (37.34) | 20 (35.09) |  |
| Comorbidity count, n (%) | |  |  |  |  | <0.001 |
| 0 | 70 (34.83) | 256 (47.32) | 29 (19.46) | 47 (25.97) | 20 (30.77) |  |
| 1 | 68 (33.83) | 177 (32.72) | 38 (25.5) | 60 (33.15) | 30 (46.15) |  |
| 2 | 39 (19.40) | 81 (14.97) | 54 (36.24) | 47 (25.97) | 9 (13.85) |  |
| 3+ | 24 (11.94) | 27 (4.99) | 28 (18.79) | 27 (14.92) | 6 (9.23) |  |
| Index of Multiple Deprivation, n (%) | | |  |  |  | 0.184 |
| Quintile 1 (most deprived) | 66 (32.84) | 143 (26.43) | 54 (36.24) | 47 (25.97) | 18 (27.69) |  |
| Quintile 2 | 52 (25.87) | 105 (19.41) | 32 (21.48) | 41(22.65) | 10 (15.38) |  |
| Quintile 3 | 35 (17.41) | 123 (22.74) | 24 (16.11) | 36 (19.89) | 16 (24.62) |  |
| Quintile 4 | 28 (13.93) | 91 (16.82) | 26 (17.45) | 34 (18.78) | 10 (15.38) |  |
| Quintile 5 (least deprived) | 20 (9.95) | 79 (14.60) | 13 (8.72) | 23 (12.71) | 11 (16.92) |  |

**Supplementary Table S7. Multinomial logistic regression model for association between patients’ characteristics and different clusters.**

|  | Clusters of similar care sequences | | | |
| --- | --- | --- | --- | --- |
|  | (Reference cluster is Low consultation and healthcare use) | | | |
|  | Increasing consultation and analgesia | High consultation and healthcare use | Low consultation but high analgesia | Low consultation but moderate healthcare use |
|  | OR (95% CI) | OR (95% CI) | OR (95% CI) | OR (95% CI) |
| Gender |  |  |  |  |
| Male | 1 | 1 | 1 | 1 |
| Female | 1.47 (1.02, 2.12) | **2.45 (1.55, 3.86)** | **2.43 (1.61, 3.67)** | **1.99 (1.08, 3.66)** |
| Age-group |  |  |  |  |
| 18-34 years | 1 | 1 | 1 | 1 |
| 35-44 years | 0.82 (0.36, 1.91) | 1.22 (0.35, 4.32) | 2.59 (0.53, 12.59) | 2.45 (0.50, 11.88) |
| 45-54 years | 1.58 (0.74, 3.38) | **3.34 (1.07, 10.47)** | **4.61 (1.02, 20.89)** | 1.89 (0.38, 9.29) |
| 55-64 years | 1.38 (0.65, 2.94) | **3.40 (1.09, 10.58)** | **7.41 (1.68, 32.66)** | 1.99 (0.42, 9.48) |
| 65-74 years | 1.81 (0.84, 3.86) | **4.79 (1.54, 14.89)** | **10.09 (2.29, 44.45)** | 3.04 (0.64, 14.32) |
| 75-84 years | 1.74 (0.75, 4.04) | **4.99 (1.51, 16.48)** | **20.89 (4.67, 93.41)** | 4.63 (0.95, 22.66) |
| 85+ years | 1.64 (0.46, 5.85) | 4.14 (0.89, 19.34) | **14.79 (2.72, 80.39)** | 4.38 (0.61, 31.15) |
| BMI |  |  |  |  |
| Underweight/Normal (<25) | 1 | 1 | 1 | 1 |
| Overweight (25-29.9) | 1.14 (0.69, 1.89) | 0.86 (0.48, 1.57) | 0.95 (0.59, 1.55) | 1.57 (0.75, 3.27) |
| Obese (≥30) | **1.93 (1.19, 3.12)** | **1.80 (1.05, 3.08)** | 1.09 (0.67, 1.78) | 1.34 (0.61, 2.94) |
| Comorbidity count | |  |  |  |
| 0 | 1 | 1 | 1 | 1 |
| 1 | 1.39 (0.94, 2.06) | **1.89 (1.10, 3.22)** | **1.88 (1.20, 2.94)** | **2.20 (1.19, 4.06)** |
| 2 | **1.61 (1.00, 2.60)** | **5.32 (3.11, 9.09)** | **2.86 (1.73, 4.71)** | 1.33 (0.57, 3.08) |
| 3+ | **2.70 (1.43, 5.09)** | **7.86 (3.94, 15.68)** | **5.15 (2.65, 9.96)** | 2.74 (0.97, 7.72) |
| Index of Multiple Deprivation | | |  |  |
| Quintile 1 (most deprived) | 1.73 (0.95, 3.16) | 2.02 (0.98, 4.15) | 1.43 (0.77, 2.66) | 0.98 (0.42, 2.29) |
| Quintile 2 | **1.84 (1.00, 3.39)** | 1.59 (0.75, 3.36) | 1.46 (0.78, 2.72) | 0.70 (0.28, 1.76) |
| Quintile 3 | 1.02 (0.54, 1.91) | 0.99 (0.46, 2.15) | 0.80 (0.43, 1.52) | 0.78 (0.34, 1.80) |
| Quintile 4 | 1.11 (0.57, 2.15) | 1.54 (0.71, 3.32) | 1.19 (0,63, 2.27) | 0.73 (0.29, 1.83) |
| Quintile 5 (least deprived) | 1 | 1 | 1 | 1 |

Significant results are highlighted in bold. OR- Odds Ratio, BMI- Body Mass Index

**Supplementary Table S8. Longitudinal linear mixed model to assess the association between clusters of similar care sequence and MSK-HQ score. Reference cluster is low consultation and healthcare use.**

|  | MSK-HQ Score | |
| --- | --- | --- |
|  | Coefficients (95% CI) | P-value |
| Fixed effects |  |  |
| Intercept | **27.65 (23.54, 31.76)** | **<0.001** |
| Cluster of similar care sequences | |  |
| Increasing consultation and analgesia | **-4.18 (-5.76, -2.60)** | **<0.001** |
| High consultation and healthcare use | **-8.63 (-10.47, -6.79)** | **<0.001** |
| Low consultation but high analgesia | **-6.18 (-7.88, -4.48)** | **<0.001** |
| Low consultation but moderate healthcare use | -1.96 (-4.49, 0.0.57) | 0.129 |
| Time |  |  |
| 3-Months | **5.26 (4.43, 6.08)** | **<0.001** |
| 6-Months | **6.27 (5.32, 7.22)** | **<0.001** |
| Interaction terms cluster of similar care sequence*time | | |
| Increasing consultation and analgesia*3-Months | **-1.75 (-3.31, -0.20)** | **0.027** |
| Increasing consultation and analgesia*6-Months | -1.05 (-2.84, 0.74) | 0.248 |
| High consultation and healthcare use*3-Months | **-4.46 (-6.15, -2.77)** | **<0.001** |
| High consultation and healthcare use*6-Months | **-4.35 (-6.31, -2.40)** | **<0.001** |
| Low consultation but high analgesia*3-Months | **-3.05 (-4.63, -1.47)** | **<0.001** |
| Low consultation but high analgesia*6-Months | **-3.63 (-5.46, -1.80)** | **<0.001** |
| Low consultation but moderate healthcare use*3-Months | -1.72 (-4.31, 0.86) | 0.192 |
| Low consultation but moderate healthcare us*6-Months | -2.44 (-5.32, 0.43) | 0.096 |
| Random effects |  |  |
| Intercept (SD) | 7.44 |  |
| Time (SD) | 2.56 |  |

Model was controlled for gender, age, BMI, comorbidity count, and index of multiple deprivation. Significant results are highlighted in bold.

**Supplementary Table S9. Difference in MSK-HQ score from low consultation and healthcare use at baseline, 3-months, and 6-months.**

|  | MSK- HQ score (Reference cluster is Low consultation and  healthcare use) | | |
| --- | --- | --- | --- |
|  | Baseline | 3-Months | 6- Months |
|  | Difference (95% CI) | Difference (95% CI) | Difference (95% CI) |
| Increasing consultation and analgesia | **-4.18 (-5.76, -2.60)** | **-5.94 (-7.86, -4.01)** | **-5.23 (-7.43, -3.04)** |
| Low consultation and healthcare use | 0 | 0 | 0 |
| High consultation and healthcare use | **-8.63 (-10.47, -6.79)** | **-13.09 (-15.26, -10.92)** | **-12.99 (-15.46, -10.52)** |
| Low consultation but high analgesia | **-6.18 (-7.88, -4.48)** | **-9.23 (-11.22, -7.24)** | **-9.82 (-12.10, -7.53)** |
| Low consultation but moderate healthcare use | -1.96 (-4.49, 0.57) | **-3.68 (-6.82, -0.55)** | **-4.40 (-7.91, -0.90)** |

Model was controlled for gender, age, BMI, comorbidity count, and index of multiple deprivation. Significant results are highlighted in bold.

**References:**

1. Raab M, Struffolino E. Sequence Analysis. SAGE Publications, Inc; 2022.

2. Vanasse A, Courteau J, Courteau M, Benigeri M, Chiu YM, Dufour I, et al. Healthcare utilization after a first hospitalization for COPD: a new approach of State Sequence Analysis based on the “6W” multidimensional model of care trajectories. BMC Health Serv Res [Internet] 2020 [cited 2022 Dec 15];20. Available from: /pmc/articles/PMC7059729/

3. Studer M. WeightedCluster Library Manual: A practical guide to creating typologies of trajectories in the social sciences with R. LIVES Working Papers 2013; 024:1–32.
